# YopN and TyeA Hydrophobic Contacts Required for Regulating Ysc-Yop Type III Secretion Activity by *Yersinia pseudotuberculosis*

**DOI:** 10.3389/fcimb.2016.00066

**Published:** 2016-06-21

**Authors:** Ayad A. A. Amer, Jyoti M. Gurung, Tiago R. D. Costa, Kristina Ruuth, Anton V. Zavialov, Åke Forsberg, Matthew S. Francis

**Affiliations:** ^1^Department of Molecular Biology, Umeå UniversityUmeå, Sweden; ^2^Umeå Centre for Microbial Research, Umeå UniversityUmeå, Sweden; ^3^Department of Molecular Biology, Uppsala BioCenter, Swedish University of Agricultural SciencesUppsala, Sweden; ^4^Joint Biotechnology Laboratory, Department of Chemistry, University of TurkuTurku, Finland; ^5^Laboratory for Molecular Infection Medicine Sweden, Umeå UniversityUmeå, Sweden

**Keywords:** protein-protein interaction, molecular modeling, protein secretion, mutagenesis, bacterial pathogenesis, regulation

## Abstract

*Yersinia* bacteria target Yop effector toxins to the interior of host immune cells by the Ysc-Yop type III secretion system. A YopN-TyeA heterodimer is central to controlling Ysc-Yop targeting activity. A + 1 frameshift event in the 3-prime end of *yopN* can also produce a singular secreted YopN-TyeA polypeptide that retains some regulatory function even though the C-terminal coding sequence of this YopN differs greatly from wild type. Thus, this YopN C-terminal segment was analyzed for its role in type III secretion control. Bacteria producing YopN truncated after residue 278, or with altered sequence between residues 279 and 287, had lost type III secretion control and function. In contrast, YopN variants with manipulated sequence beyond residue 287 maintained full control and function. Scrutiny of the YopN-TyeA complex structure revealed that residue W_279_ functioned as a likely hydrophobic contact site with TyeA. Indeed, a YopN_*W*279*G*_ mutant lost all ability to bind TyeA. The TyeA residue F_8_ was also critical for reciprocal YopN binding. Thus, we conclude that specific hydrophobic contacts between opposing YopN and TyeA termini establishes a complex needed for regulating Ysc-Yop activity.

## Introduction

Human pathogenic *Yersinia* are represented by the species *Yersinia pestis*, the causative agent of bubonic, septicemic, and pneumonic plague (Zhou et al., 2006), as well as *Yersinia enterocolitica* and *Yersinia pseudotuberculosis* that are responsible for mild self-limiting gastrointestinal infections that are rarely systemic (Naktin and Beavis, 1999). A common virulence strategy among these bacteria is the plasmid-borne Ysc-Yop type III secretion system (T3SS; Portnoy et al., 1984; Cornelis et al., 1998). This confers to *Yersinia* a tropism for immune cell rich lymphatic tissue where they resist phagocytosis to preferentially remain in an extracellular replicative niche (Fällman and Gustavsson, 2005). Numerous other Gram-negative bacteria also employ T3SSs to establish parasitic or mutualistic interactions with eukaryotic hosts (Pallen et al., 2005b; Troisfontaines and Cornelis, 2005). Most T3SSs consist of 20–25 proteins that assemble into a hollow protein transport channel traversing the bacterial envelope and protruding out from the bacterial surface to connect with target eukaryotic cells. Through this portal cytoplasmic anti-host effectors can be injected direct to the host cell interior in a one-step process, or surface-located proteins can be delivered into the eukaryotic cell via a two-step process (Edgren et al., 2012).

Assembly is a coordinated process involving the build-up of different sub-parts that eventually connect to form one coherent structure (Kosarewicz et al., 2012). Assembly may either start in the inner membrane and build from the inside-out (Schraidt et al., 2010; Wagner et al., 2010), or it may begin with the simultaneous formation of structures in both the inner and outer membranes (Diepold et al., 2011). When completed, a universal specificity switch mechanism involving auto-processing of the inner membrane embedded YscU family of homologous proteins dictates the secretion of needle components followed by the distal needle tip proteins and the hydrophobic translocator proteins that dock with the host cell to form a translocon pore in the plasma membrane (Frost et al., 2012; Hughes, 2012).

Less defined are mechanisms that delay effector protein secretion until the translocon pore has assembled. Almost all secreted substrates require a dedicated T3S chaperone to prevent premature protein interactions in the cytoplasm and also to ensure their efficient secretion (Francis, 2010). In some cases, the secretion of hydrophobic translocators allows their free cognate T3S chaperone to act as a cofactor to induce subsequent transcription of effector genes (Darwin and Miller, 2001; Mavris et al., 2002; Pilonieta and Munson, 2008). A number of studies also propose mechanisms for enhancing the secretion efficiency of translocator proteins over effector proteins. This can involve recognition of their customized chaperones by a cytoplasmic sorting platform that comprises a complex of the SpaO (FliN/HrcQ/Spa33/YscQ), OrgA (HrpD/MxiK/YscK), and OrgB (FliH/HrpE/MxiN/YscL) protein families (Lara-Tejero et al., 2011). It could also involve free translocator T3S chaperone interacting with the FliJ family of proteins at the cytoplasmic face of the inner membrane to improve their ability to reload with their translocator cargo and expedite secretion (Evans and Hughes, 2009). In addition, specific sequences within the translocator proteins may have evolved into distinctive secretion signals that are preferentially recognized by the T3SS to prioritize their secretion (Munera et al., 2010; Amer et al., 2011; Tomalka et al., 2012). In other cases, this recognition might occur via direct interaction with members of the InvE family of proteins (Kubori and Galán, 2002; Kim et al., 2013). Some members of this protein family also bind effector substrates to delay their secretion (O'Connell et al., 2004; Deng et al., 2005; Wang et al., 2008) or even to the system ATPase at the base of the T3SS channel to physically block effector secretion (Botteaux et al., 2009; Martinez-Argudo and Blocker, 2011; Cherradi et al., 2013).

In the Ysc-Yop T3SS of *Yersinia*, YopN, and TyeA possess homology to the N- and C-terminus of InvE-like proteins, respectively (Pallen et al., 2005a). Consistent with this homology, a complex of YopN and TyeA, in cooperation with the cognate YopN secretion pilot chaperone composed of a SycN and YscB heterodimer, control substrate secretion by plugging the secretion channel (Forsberg et al., 1991; Day and Plano, 1998; Jackson et al., 1998; Iriarte and Cornelis, 1999; Cheng and Schneewind, 2000; Cheng et al., 2001; Ferracci et al., 2005; Schubot et al., 2005; Joseph and Plano, 2013). The significance of this secretion control function is reflected in the deregulated secretion profiles exhibited by bacterial strains harboring full length deletions of the *yopN* and/or *tyeA* alleles (Forsberg et al., 1991; Day and Plano, 1998; Iriarte et al., 1998; Jackson et al., 1998; Cheng et al., 2001; Lee et al., 2001; Sundberg and Forsberg, 2003; Ferracci et al., 2004, 2005; Amer et al., 2013). Until recently it was not known how the YopN-TyeA complex tethers to the T3S apparatus to plug the export channel. Now it has been revealed that Pcr1, the TyeA homolog in *Pseudomonas aeruginosa*, complexes with PcrG (LcrG in *Yersinia*) and then co-assembles with the integral inner membrane protein PcrD (YscV) to block access of substrates to the secretion channel (Lee et al., 2014).

**Figure 1:**
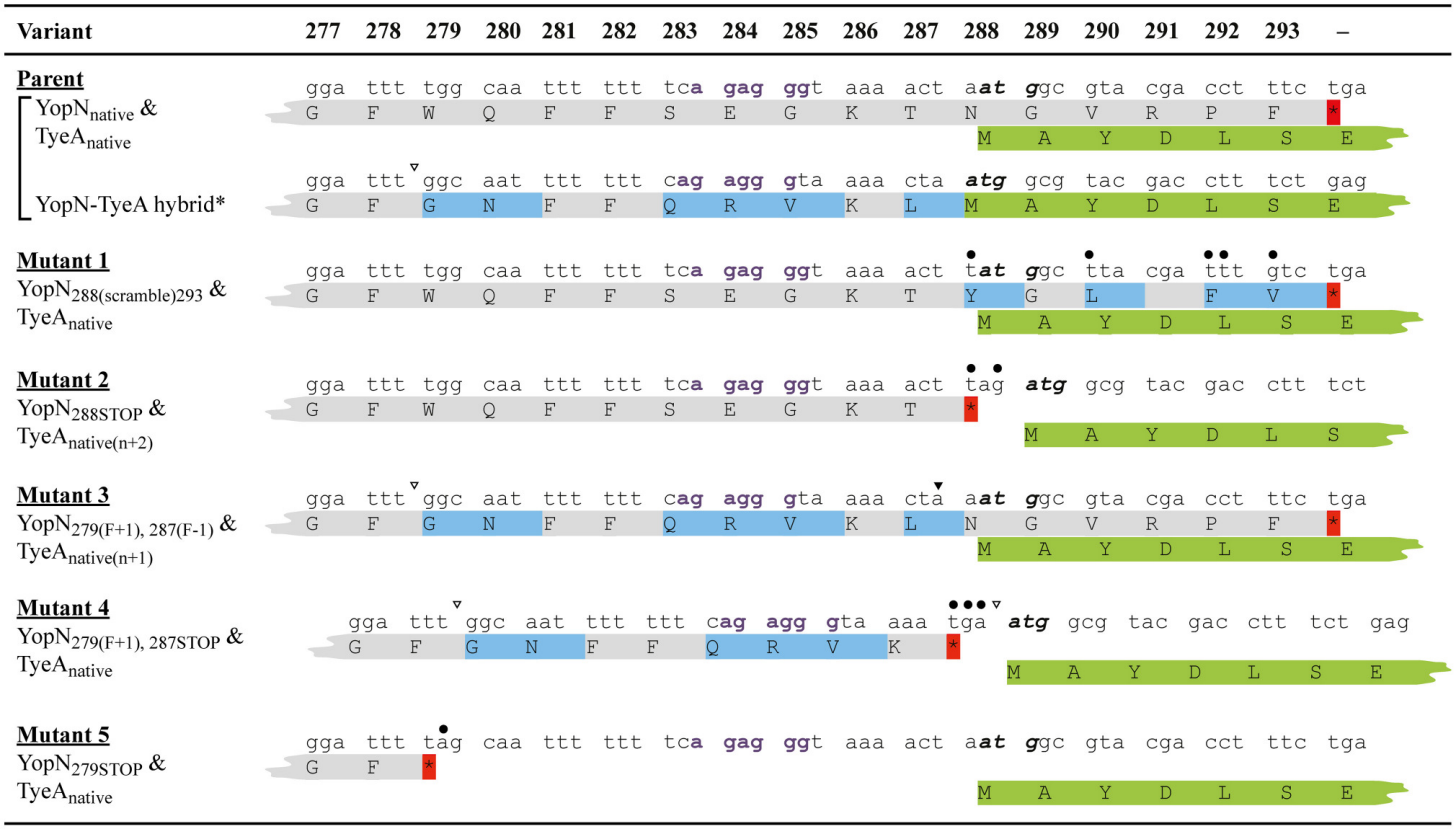
**A comparison of the nucleotide and amino acid sequence changes in the key ***in cis yopN*** mutations used in this study**. Shown is nucleotide (lower case font) and amino acid (upper case font; single letter code) sequence encompassing codon positions 277–293 of YopN and the overlapping 1–7 codons of TyeA. Derived from the native sequence (Parent), three different polypeptides can be generated—YopN_native_, TyeA _native_, and a YopN-TyeA hybrid fusion product resulting from an unconfirmed +1 frameshift mutation after codon 279 (Ferracci et al., 2004; Amer et al., 2013). Shading in light gray indicates the YopN_native_ amino acid sequence. Amino acids shaded in light blue are YopN sequences that differ from the native protein due to a natural or engineered alteration to the codon sequence. Introduced site-directed nucleotide substitutions are highlighted by an overlying filled-in circle. Open arrowheads above the nucleotide sequence specifically locate positions of nucleotide deletions that result in a +1 frameshift, and filled-in arrowheads identify nucleotide insertions that serve as compensatory −1 frameshifts. No mutation altered the coding sequence of overlapping *tyeA* as shown by routine retention of the first 6–7 TyeA residues in green (TyeA_native_); the start codon of which is highlighted in bold italic font. However, bacteria producing Mutant 2 (YopN_288STOP_) and Mutant 3 [YopN_279(F+1), 287(F−1)_] have a displaced *tyeA* initiation codon relative to a putative Shine-Dalgarno sequence (“agaggg” in bold purple font) by n + 2 [TyeA_native(n+2)_] and n + 1 [TyeA_native(n+1)_], respectively. Also note that in these bacteria and in bacteria producing Mutant 4 [YopN_279(F+1), 287STOP_], *tyeA* coding sequence assumes a different reading from the native sequence. Native or introduced *yopN* termination codons are indicated by an asterisk (red shade). Two additional mutations were genetically engineered and are designated Mutant 1 (YopN_288(scramble)293_) and Mutant 5 (YopN_279STOP_).

Curiously, YopN and TyeA can be synthesized as a singular YopN-TyeA polypeptide (Ferracci et al., 2004; Amer et al., 2013). Probably this occurs via transcriptional strand slippage to introduce a +1 frameshift after codon 278 of *yopN* that contributes to YopN-TyeA hybrid production, although this is not yet experimentally verified (Figure 1; Ferracci et al., 2004; Amer et al., 2013). In all three *Yersinia* species known to be pathogenic to humans, the *yopN* DNA sequence where the frameshift is believed to occur contains stretches of T's that may contribute to strand slippage. Despite this, some strains of *Y. enterocolitica* do not produce a natural hybrid of YopN and TyeA, most likely because of a defined single nucleotide difference that would place a TAA termination codon upstream of *tyeA* following a + 1 frameshift event (Ferracci et al., 2004). Hence, on the basis of these anomalies it is unclear whether the YopN-TyeA hybrid has evolved a role in *Yersinia* T3SS function. Mutants of *Y. pseudotuberculosis* designed to produce only the YopN-TyeA hybrid alone maintained *in vitro* low Ca^2+^-dependent control of substrate T3S, but were unable to control fully the polarized translocation of effectors into the cytosol of eukaryotic cells, and this impinged on their ability to survive *in vivo* infections of mice (Amer et al., 2013). The non-functional hybrids contained a C-terminal YopN sequence beyond residue 278 that barely resembled native YopN. In this study, scrutiny of this C-terminal region revealed a small segment necessary for full YopN function, within which was the W_279_ residue that specifically established hydrophobic contacts with the N-terminus of TyeA to maintain Ysc-Yop regulatory control.

## Materials and methods

### Bacterial strains and growth conditions

Bacterial strains used in this study are listed in electronic Supplementary Material, Table S2. Bacteria were routinely cultivated in Luria Bertani (LB) agar or broth at either 26°C (*Y. pseudotuberculosis*) or 37°C (*E. coli*) with aeration. Where required, appropriate antibiotics were added at the final concentrations of carbenicillin (Cb; 100 μg per ml), kanamycin (Km; 50 μg per ml) and chloramphenicol (Cm; 25 μg per ml).

### PCR amplification and sequence analysis

Amplified DNA fragments were obtained by PCR using the various oligonucleotide combinations listed in electronic Supplementary Material (Table S3), which were earlier synthesized by Sigma-Aldrich Co (Dorset, England). All amplified DNA fragments where quality controlled by sequence analysis (Eurofins MWG Operon AG, Ebersberg, Germany) of clones generated using the InsTAclone PCR cloning kit (Thermo Fisher Scientific, Inc.).

### Construction of *yopN* and *tyeA* mutations

Various site-directed and deletion mutations in the *yopN* and *tyeA* alleles were first generated by the classical two-step overlap PCR procedure. For analysis of mutated alleles *in trans*, PCR amplified and sequenced DNA fragments were cloned directly into appropriate expression vectors. To generate *in cis* mutations of *yopN* or *tyeA*, sequence-confirmed DNA fragments were subsequently cloned into the SalI-XbaI digested suicide mutagenesis vector, pDM4 (a gift from Debra Milton; electronic Supplementary Material, Table S2), and using *E. coli* S17-1λ*pir* as the donor in conjugal matings, were then transferred into parental *Y. pseudotuberculosis* (YPIII/pIB102). Allelic exchange of the virulence plasmid-encoded wild type *yopN* or *tyeA* copy with individual *yopN* or *tyeA* mutations was selected for using conventional *sacB*-mediated sensitivity to 5% sucrose. Mutants were confirmed by a combination of diagnostic PCR and sequence analysis.

### Protein stability

To measure stability of accumulated cytoplasmic YopN or TyeA exposed to endogenous proteases, *de novo* protein synthesis was inhibited by the addition of 50 μg/ml chloramphenicol prior to sample collection as described previously (Feldman et al., 2002).

### Type III secretion substrate synthesis and secretion

Analysis of T3SS by *Y. pseudotuberculosis* was performed according to standard protocol (Amer et al., 2011) after growth at 37°C in Brain heart infusion (BHI) broth. Media containing Ca^2+^ ions was the non-inducing condition (BHI supplemented with 2.5 mM CaCl_2_), while media devoid of Ca^2+^ ions was the inducing condition (BHI supplemented with 20 mM MgCl_2_ and 5 mM Ethylene glycol-bis-(β-aminoethyl ether)-N,N,N′,N′-tetraacetic acid). Total protein associated with whole bacterial culture was assessed by sampling direct from the bacterial suspension. Sampling of the cleared supernatant provided an assessment of the secreted protein levels. All protein fractions were separated by SDS-PAGE and subjected to immunoblotting using the semi-dry transfer technique onto PDVF membranes. Detection of *Yersinia* substrates used rabbit polyclonal antisera raised against the secreted YopN, YopD, and YopE (a gift from Hans Wolf-Watz) or non-secreted TyeA (a gift from Gregory Plano), an anti-rabbit antibody conjugated to horseradish peroxidase, and chemiluminescent detection with the Pierce ECL 2 Western Blotting Substrate.

### Assessment of T3S activity in the presence of eukaryotic cells

To indirectly assess the efficiency of the Ysc-Yop T3SS to translocate effectors into eukaryotic cells we measured the viability of *Yersinia* in the presence of murine macrophage-like J774 cells (Bartra et al., 2001; Amer et al., 2011, 2013; Costa et al., 2012, 2013). This assay capitalizes on the anti-phagocytic properties of the Ysc-Yop T3SS. Bacteria lacking a fully functional T3SS are therefore more efficiently phagocytosed and these intracellular bacteria are susceptible to the antimicrobial killing effects of J774 cells. This assay tests the total recovery of bacteria associated with host cells, which includes both surface attached and intracellular bacteria. Hence any reduction in bacterial viability as determined by CFU counts reflects the amount of bacteria that were susceptible to immune cell killing following phagocytosis.

### Plasmid construction, transformation, and yeast two-hybrid analysis

To facilitate YopN and TyeA interaction studies in yeast, wild type and mutated *yopN* alleles were cloned into the EcoRI/*Bam*HI restricted GAL4 DNA-binding domain plasmid pGBKT7 (Clontech Laboratories, Palo Alto, CA, USA), while wild type and mutated *tyeA* alleles were cloned into the EcoRI/*Bam*HI restricted the GAL4 activation domain plasmid pGADT7 (Clontech Laboratories). Transformation of the *Saccharomyces cerevisiae* reporter strain AH109 and analysis of protein-protein interactions was performed as described in detail earlier (Francis et al., 2000). Verification of protein stability by isolation and analysis of yeast protein extracts has also been described (Francis et al., 2000).

### Cysteine cross-linking

*In vivo* disulphide cross-linking was performed as essentially described previously (Lee et al., 2006; Gueguen et al., 2011), but with some slight modifications. Briefly, *Yersinia* bacteria containing engineered YopN and TyeA with strategically placed cysteine substitutions were grown in inducing condition (BHI supplemented with 20 mM MgCl2 and 5 mM EDTA). Cells were harvested by centrifugation and washed with 10 ml of 20 mM sodium phosphate (NaP) buffer, pH 6.8 [20.29 mM NaH_2_PO_4_.H_2_O (monobasic), 19.57 mM Na_2_HPO_4_ (dibasic)]. After washing, the cells were resuspended in 1.6 ml of NaP and aliquoted into three samples of 300 μl each. For a control, cells were incubated only with buffer. For the oxidized sample, cells were treated with 0.3 mM dichloro(1,10-phenanthroline) copper(II; Cu-oP; Sigma-Aldrich) for 20 min at room temperature. The reaction was subsequently quenched by addition of 2.5 mM N-ethyl-maleimide (NEM; Sigma-Aldrich) for 15 min at room temperature to quench the reaction. To the reduced sample was added 0.3 mM Cu-oP and 2.5 mM NEM simultaneously, centrifuged and resuspended in SDS-PAGE sample buffer containing 10 mM DTT as reducing agent. After centrifugation of the control and the oxidized samples, they were resuspended in SDS-PAGE sample buffer without the DTT reducing agent.

### Structure modeling and analysis

The model of the YopN-TyeA fusion protein was constructed based on the crystal structure of the YopN-TyeA complex (RCSB PDB accession code 1XL3; Schubot et al., 2005) using program O (Jones et al., 1991). The connecting loop was designed based on search of the loop library, keeping high restrains for stereochemistry. The side chains of residues at the C-terminus that are altered due to the +1 frame-shift were modeled using the most frequently found rotamer conformations. The interactive surfaces were analyzed using the AREAIMOL program from the CCP4 crystallography suite (CCP4, 1994).

### Statistics

An unpaired *t*-test with Welch's correction performed by means of GraphPad Prism version 5.00 for Windows, GraphPad Software, San Diego California USA, www.graphpad.com was used to analyse the differences in data sets. Differences with a probability value of *P* < 0.05 were considered significant.

### Ethics statement

Infection studies were performed in strict accordance with the Swedish Bioethical Guidelines for care and use of laboratory animals. The protocol was approved by the Umeå Committee on the Ethics of Animal Experiments (Permit Number: A-60-10).

## Results

### Site-directed mutagenesis of the YopN C-terminus

Genetically engineered YopN-TyeA hybrids were compromised for Ysc-Yop T3SS activity in the presence of host cells and in the mouse infection model (Amer et al., 2013). As these were constructed via an introduced +1 frameshift mutation that caused altered coding potential in *yopN* after codon 278, it suggested that the extreme YopN C-terminus might be needed for proper T3S activity in *Y. pseudotuberculosis* (Amer et al., 2013). To investigate this, we generated five site-directed mutations localized within the 3-prime end of *yopN* (Table 1). To avoid any copy number effects, mutated versions of the *yopN* gene were used to replace the wild type allele on the virulence plasmid in *Yersinia*.

**Table 1:** **Summation of phenotypes exhibited by strains with ***in cis*** mutations in ***yopN*** and ***tyeA*****.

**Variant**	**Stability^a^**	**Growth^b^**	**Synthesis and secretion**			**Viability^e^**	**Virulence attenuation^f^**	**TyeA binding^g^**	
			**Surface YscF^c^**	**YopN (Hybrid)^d^**	**Other Yops^d^**			**YTH**	**BACTH**
YopN_288(scramble)293_	WT	WT	WT	WT (WT)	WT	WT	WT	WT	WT
YopN_288STOP_	WT	WT	WT	WT (↓)	WT	Null-like	WT	WT	WT
YopN_279(F+1), 287(F−1)_	↓	Null	WT	Null (↓↓)	Null	Null	ND	Null	Null
YopN_279(F+1), 287STOP_	WT	Null	WT	Null-like (↓)	Null-like	Null-like	ND	Null	Null^*^
YopN_279STOP_	(↓)	Null	WT	Null (↓)	Null	Null	ND	Null	Null^*^
YopN_W279G_	↓	Null	WT	Null (↑)	Null	Null-like	ND	Null	Null^*^
TyeA_Y3A_	WT	WT	WT	WT (WT)	WT	WT	ND	WT	WT
TyeA_L5A_	(↓)	WT	WT	WT (WT)	WT	WT	ND	WT	WT
TyeA_F8A_	↓	Null	WT	Null (↑)	Null	Null-like	ND	Null	Null^*^
TyeA_F33A_	↓	Null-like	WT	Null-like (WT)	Null-like	Null-like	ND	WT-like	Null^*^

a *A summary of the intrabacterial stability of each YopN and TyeA variant shown in Figure 4 and as determined by the method of Feldman et al. (2002). WT: normal stability; (↓): slight instability; ↓: moderate instability*.

b *Analysis of growth Y. pseudotuberculosis phenotypes was performed as previously described (Amer et al., 2011, 2013). Results shown in electronic Supplementary Material, Figure S1 are summarized as wild type (WT) that represents the phenotype of parental bacteria (YPIII/pIB102) or conversely as “Null” that represents the single ΔyopN or ΔtyeA null mutants or the double ΔyopN, tyeA null mutant. “WT” growth refers to calcium dependency (CD) at 37°C and reflects wild type regulatory control of Yop synthesis by virtue of a functional YopN-TyeA regulatory complex, whereas “Null” growth refers to temperature sensitivity (TS) at 37°C and echoes defective regulatory control whereby Yop synthesis is constitutive due to a defective YopN–TyeA regulatory complex (Iriarte et al., 1998; Cheng et al., 2001; Schubot et al., 2005). Null-like reflects a growth phenotype the lies between CD and TS, where bacteria grow only modestly at 37°C in the presence of calcium*.

c *Analysis of cross-linked YscF higher-order structures derived from the bacterial surface was used to gage if Ysc T3SS's are correctly assembled and competent for Yops substrate secretion (Amer et al., 2013). Results shown in electronic Supplementary Material, Figure S2 are summarized as like wild type (WT) or the ΔyscU, lcrQ null mutant (Null)*.

d *A summary of the degree of controlled Yop synthesis and secretion generated from bacterial strains harboring the yopN and tyeA mutations as determined for production of YopN (Figures 2A, 7A) as well as the YopD injectisome component and the injected YopE cytotoxic effector (Figures 2B, 7B). WT: normal substrate synthesis and secretion in inducing conditions; Null: deregulated (constitutive) Yops synthesis and secretion; Null-like: partial deregulation. In parenthesis is an assessment of YopN-TyeA hybrid formation (Figures 2A, 7A). WT: normal formation; ↓: low level formation; ↓↓: not readily detectable by standard immunoblot; ↑: deregulated (constitutive) YopN-TyeA hybrid synthesis and secretion*.

e *As a gage for measuring the effectiveness of Ysc-Yop T3SS activity, we analyzed the degree in which Yersinia could resist engulfment by professional phagocytic cells and subsequent intracellular killing by host antimicrobial activities (Bartra et al., 2001; Amer et al., 2011, 2013; Costa et al., 2012, 2013). The results are a summary of data presented in Figure 3. WT: bacteria maintain a high degree of viability being indistinguishable from wild type; Null-like: the ability to maintain viability is impaired; Null: bacteria are as susceptible to immune cell killing as is the ΔyopN null mutant*.

f *Groups of five mice were co-infected with a the parental strain and strains containing yopN mutated alleles. The degree of attenuation was determined by competitive index measurements as detailed in electronic Supplementary Material, Table S1 and previously (Amer et al., 2013). WT: virulence of mutant bacteria was not statistically different from the parent; ND, not determined*.

g Determined from conventional yeast two-hybrid assay (YTH; Figure 5; Francis et al., 2000) and bacterial adenylate cyclase two hybrid (BACTH; electronic Supplementary Material, Figure S3; Thanikkal et al., 2012). WT: robust interaction between YopN and TyeA; Null: no detectable binding between YopN and TyeA; WT-like: a modest interaction between YopN and TyeA. The asterisk (^*^) indicates that one or both fusion proteins were unstable or not detected by immunoblot analysis.

One set of mutants targeted the six codon overlapping region between the YopN C-terminus and the TyeA N-terminus (Figure 1). The first mutation scrambled all possible nucleotides in the codon wobble position to specifically alter the C-terminal codon potential of YopN only, thereby generating a YopN_288(scramble)293_ variant (Mutant 1). The second mutation introduced the “TAG” stop codon after *yopN* codon 287, which gave rise to bacteria producing YopN_288STOP_ that lacked the extreme C-terminal residues 288–293 (Mutant 2).

A second set of mutants was focused on the region of YopN incorporating residues 279–287 (Figure 1). The first of these, YopN_279(F+1), 287(F−1)_, contained the same +1 frameshift deletion after codon 278 that was followed by a compensatory insertion of an “A” nucleotide to restore the reading frame after codon 287 (Mutant 3). The second of these, YopN_279(F+1), 287STOP_, was constructed by a +1 frameshift in which a “T” nucleotide was deleted immediately after codon 278 followed by the insertion of a stop codon “TGA” in place of codon 287 (Mutant 4). The third mutant of these, YopN_279STOP_, was generated via the introduction of the “TAG” stop codon after residue 278 resulting in YopN lacking the C-terminal residues 279–293 (Mutant 5).

Critically, all these allelic variants left the integrity of the partially overlapping *tyeA* coding sequence intact. However, mutant 2 and mutant 3 altered the position of the putative Shine-Dalgarno sequence (“agaggg”) relative to the *tyeA* start codon from the customary 8 nucleotides to 10 nucleotides (e.g., n + 2) and 9 nucleotides (e.g., n + 1), respectively (Figure 1). We then performed a functional analysis of the YopN C-terminus using both *in vitro* and *in vivo* phenotypic assays. A summary of the YopN mutant phenotypes is provided in Table 1.

### Null phenotypes caused by mutations that disrupt the region of YopN encompassing residues 279–287

Mutants 3–5 that respectively produced the YopN_279(F+1), 287(F−1)_, YopN_279(F+1), 287STOP_, and YopN_279STOP_ variants, exhibited essentially null phenotypes with respect to *in vitro* and *in vivo* T3SS activity. We first assayed the growth phenotype of these strains, in terms of temperature-sensitivity and calcium-dependence. Typically wild type strains are unable to grow without the addition of Ca^2+^, while *yopN* and *tyeA* null mutants are temperature-sensitive, able to grow at 26°C but not at 37°C even in the presence of Ca^2+^ (electronic Supplementary Material, Figure S1; Forsberg et al., 1991; Lee et al., 1998; Cheng and Schneewind, 2000; Ferracci et al., 2005; Amer et al., 2013). Similar to these previous reports of defective YopN mutants, our three *yopN* mutant strains were severely growth restricted at elevated temperature—a growth phenotype known as temperature sensitive (electronic Supplementary Material, Figure S1, Mutants 3–5).

Temperature sensitive *Yersinia* are usually deregulated for Yop synthesis, causing constitutive protein production regardless of Ca^2+^ levels. For this *yopN* mutant set, we investigated the impact of temperature sensitivity on Yop synthesis and secretion in two ways. First, using a procedure involving chemical cross-linking and YscF immunoblots we determined the amount of the outermost YscF needle appendage assembled at the distal extremity of T3SS structures spanning the bacterial envelope of the various *yopN* mutant strains (electronic Supplementary Material, Figure S2A; Amer et al., 2013). This revealed that all three strains assembled YscF at the bacterial surface, at levels comparable to full length *yopN* null mutants, and these levels far exceeded the amounts observed for parental bacteria (electronic Supplementary Material, Figure S2A, Mutants 3–5). Second, we used a combination of fractionation and immunoblotting to measure the amount of total Yops production (in raw culture media that contains both bacteria associated Yops and freely secreted Yops) and the amount of free Yops secreted into the cleared culture supernatants of the various mutant strains grown in *in vitro* laboratory media (Figure 2). This demonstrated that the YopN_279(F+1), 287(F–1)_, YopN_279(F+1), 287STOP_ and YopN_279STOP_ variants could no longer maintain Ca^2+^-dependent control of Yops synthesis and secretion *in vitro* (Figure 2, Mutants 3–5). The extent of Yops deregulation was most severe for bacteria producing the YopN_279(F+1), 287(F–1)_ and YopN_279STOP_ variants, which mirrored the degree of deregulation caused by the complete removal of the *yopN* allele or the *tyeA* allele (Figure 2; Forsberg et al., 1991**;** Lee et al., 1998**;** Cheng and Schneewind, 2000**;** Ferracci et al., 2005**;** Amer et al., 2013). The deregulation of Yops synthesis and secretion in these strains is corroborated by the corresponding elevated levels of surface localized YscF (see Figure S2A).

**Figure 2:**
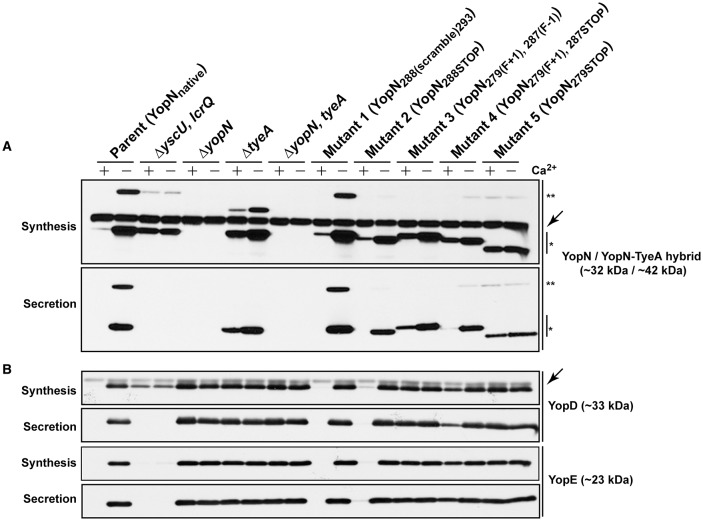
**Yop synthesis and secretion by ***in vitro*** grown ***Yersinia*****. Bacteria were grown in BHI medium either with (+) or without (−) Ca^2+^. Collected samples consisted of a mix of proteins contained within intact bacteria and associated with the outer bacterial surface that were retained in the bacterial pellet (Synthesis) or Yop proteins secreted free into the extracellular medium obtained from the cleared culture supernatants (Secretion). These were fractionated on a long 12% SDS-PAGE, wet-blotted onto PDVF membrane and then analyzed by immunoblot using polyclonal rabbit anti-YopN antiserum **(A)** or polyclonal rabbit anti-YopD and anti-YopE antiserum **(B)**. The single asterisk (^*^) highlights the singular YopN (~32 kDa) polypeptide, while the double asterisk (^**^) reveals the naturally produced and secreted ~42 kDa YopN-TyeA hybrid. The arrows (←) indicate a non-specific protein band recognized by the anti-YopN antiserum and the anti-YopD antiserum. The band appearing just above the nonspecific band in the Δ*tyeA* strain likely represents a frameshifting event that causes full-length YopN to be fused with the TyeA_Δ19−59_ deletion remnant resulting in a hybrid product that has a predicted molecular weight of ~38 kDa. Strains: Parent (YopN_native_), YPIII/pIB102; Δ*yscU, lcrQ* double mutant, YPIII/pIB75-26; Δ*yopN* null mutant, YPIII/pIB82; Δ*tyeA* null mutant, YPIII/pIB801a; Δ*yopN, tyeA* double mutant, YPIII/pIB8201a; Mutant 1–YopN_288(scramble)293_, YPIII/pIB8213; Mutant 2–YopN_288STOP_, YPIII/pIB8212; Mutant 3–YopN_279(F+1), 287(F−1)_, YPIII/pIB8208; Mutant 4–YopN_279(F+1), 287STOP_, YPIII/pIB8207; Mutant 5–YopN_279STOP_, YPIII/pIB8209. The theoretical molecular masses predicted from amino acid sequence are given in parentheses.

**Figure 3:**
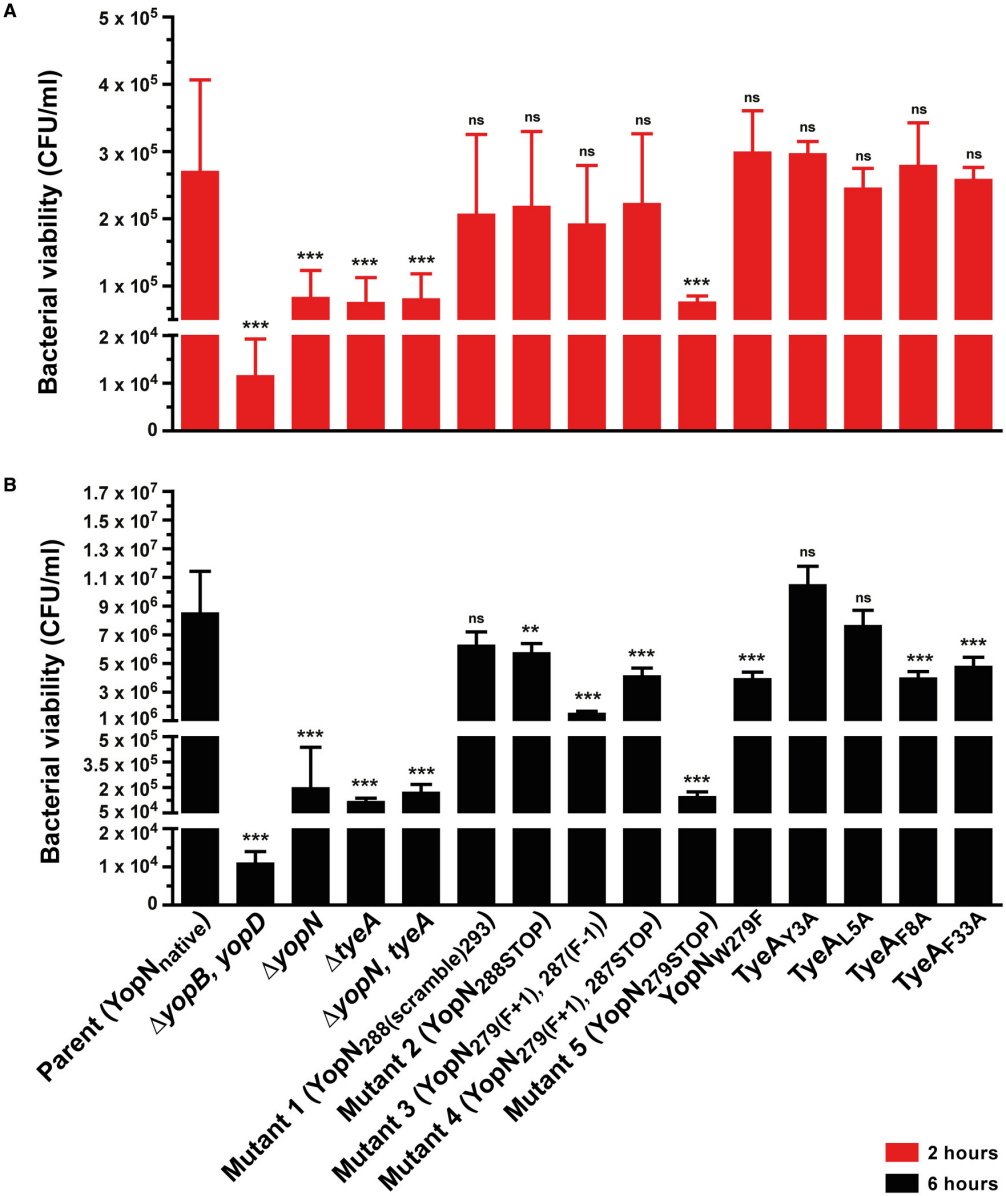
*****Yersinia*** susceptibility to killing by macrophages**. *Y. pseudotuberculosis* strains were used to infect murine macrophage-like J774-1 cells. Bacterial cells with a compromised T3SS were more rapidly phagocytosed and killed by these immune cells. Bacterial survival as measured by CFU/ml was determined at 2 h **(A)** and 6 h **(B)** post-infection. The results are expressed as a mean of at least six independent assays ± the standard deviation (Unpaired *t*-test with Welch's correction; *n* = 12–22 cells; α = 0.05; ns, not significant, *P* > 0.05; ^**^P ≤ 0.01; ^***^P ≤ 0.001). Of all the site-directed point mutants examined, bacteria producing YopN_288(scramble)293_, TyeA_Y3A_ and TyeA_L5A_ still show comparable viability to parental bacteria after 6 h, whereas Mutant 5 (YopN_279STOP_) is particularly compromised to the extent of null mutants in *yopN* or *tyeA*.. Strains: Parent (YopN_native_), YPIII/pIB102; Δ*yopB, yopD* double mutant, YPIII/pIB619; Δ*yopN* null mutant, YPIII/pIB82; Δ*tyeA* null mutant, YPIII/pIB801a; Δ*yopN, tyeA* double mutant, YPIII/pIB8201a; Mutant 1–YopN_288(scramble)293_, YPIII/pIB8213; Mutant 2–YopN_288STOP_, YPIII/pIB8212; Mutant 3–YopN_279(F+1), 287(F−1)_, YPIII/pIB8208; Mutant 4–YopN_279(F+1), 287STOP_, YPIII/pIB8207; Mutant 5–YopN_279STOP_, YPIII/pIB8209; YopN_W279G_, YPIII/pIB8223; TyeA_Y3A_, YPIII/pIB8221; TyeA_L5A_, YPIII/pIB8222; TyeA_F8A_, YPIII/pIB8220; TyeA_F33A_, YPIII/pIB8219.

Quite probably, Yops secretion into laboratory media is an *in vitro* artifact. To compensate for this, we also assessed the ability of the T3SS to permit the extracellular survival of bacteria in the presence of professional phagocyte monolayers (Figure 3; Bartra et al., 2001; Amer et al., 2011, 2013; Costa et al., 2012, 2013). Hence, deregulation of Yops synthesis and secretion was manifested in an ineffective bacterial defense against killing by immune cells *in vivo*. In particular, the bacterial mutant producing the YopN_279STOP_ form was as susceptible to immune cell killing as the full length *yopN* null mutant and the *tyeA* null mutant at both 2 and 6 h time points (Figures 3A,B, Mutant 5). Additionally at the 6 h time point, bacteria producing YopN_279(F+1), 287(F−1)_ and YopN_279(F+1), 287STOP_ were also more susceptible than parental bacteria to immune cell killing, but to a lesser degree than was observed for the full length null mutants (Figure 3B, Mutants 3 and 4). We also considered to examine the effect that Yops deregulation in this set of three mutants has on virulence attenuation in a mouse model of infection. However, studying a Δ*yopN* null mutant had earlier revealed that a temperature sensitive growth defect caused severe attenuation during competitive infections of mice; we have previously measured a competitive index (CI) of 0.00007 for this strain, which is >11000 fold less virulent than parental bacteria that displayed a CI of 0.83 (electronic Supplementary Material, Table S1; Amer et al., 2013). Consequently, we opted not to perform infection studies with these additional temperature sensitive strains harboring *yopN* mutated alleles.

**Figure 4:**
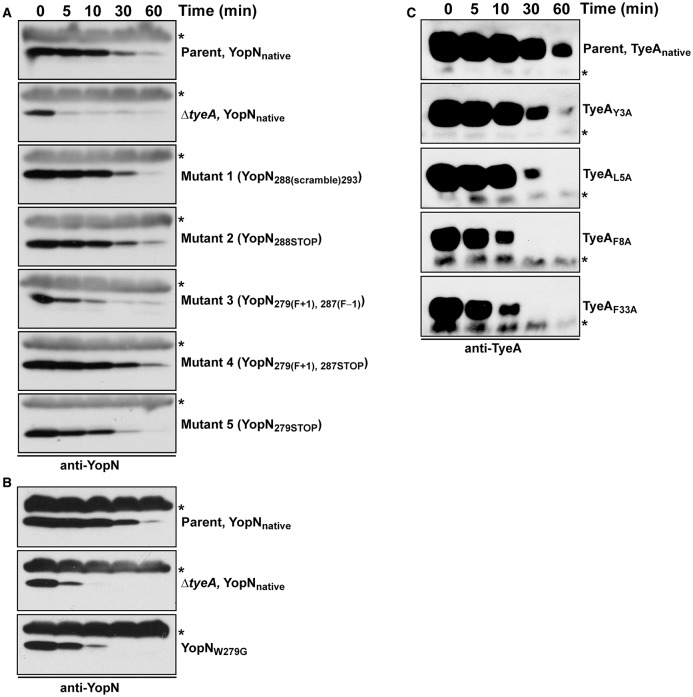
**Intrabacterial stability of pre-formed pools of YopN and TyeA variants**. Bacteria were first cultured for 1 h in non-inducing (plus 2.5 mM CaCl_2_) BHI broth at 37°C. The protein synthesis inhibitor chloramphenicol (50 μg/ml) was added at time point 0 min (min). Samples were then collected at this and subsequent time points. Protein levels associated with pelleted bacteria were detected by Western blot using rabbit polyclonal anti-YopN antiserum **(A,B)** or anti-TyeA antiserum **(C)**. Non-specific cross-reacting bands were used as convenient loading controls and are indicated by an asterisk (^*^). Parent (YopN_native_ and TyeA_native_), YPIII/pIB102; Δ*tyeA* null mutant (YopN_native_), YPIII/pIB801a; Mutant 1–YopN_288(scramble)293_, YPIII/pIB8213; Mutant 2–YopN_288STOP_, YPIII/pIB8212; Mutant 3–YopN_279(F+1), 287(F−1)_, YPIII/pIB8208; Mutant 4–YopN_279(F+1), 287STOP_, YPIII/pIB8207; Mutant 5–YopN_279STOP_, YPIII/pIB8209; YopN_W279G_, YPIII/pIB8223; TyeA_Y3A_, YPIII/pIB8221; TyeA_L5A_, YPIII/pIB8222; TyeA_F8A_, YPIII/pIB8220; TyeA_F33A_, YPIII/pIB8219.

Critically, targeting the region encoding resides 279–287 by site-directed mutagenesis did not cause a general increase in their *in vivo* susceptibility to proteolysis, at least as measured by the fact that both YopN_279(F+1), 287STOP_ and YopN_279STOP_ displayed a stability that was reminiscent of wild type protein (Figure 4, Mutants 4 and 5). However, the variant YopN_279(F+1), 287(F−1)_ did displayed some reduction in stable protein levels when compared to native YopN (Figure 4, Mutant 3). This mutant has therefore a heightened sensitivity to proteolysis.

### Disruption of the YopN-TyeA regulatory complex

Current thinking suggests that a TyeA anchor aids stable YopN to form a plug in the T3S channel that serves to prevent Yop substrate entry into the secretion channel until appropriate environmental cues such as target cell contact have been sensed and interpreted by *Yersinia* (Cheng and Schneewind, 2000; Cheng et al., 2001; Ferracci et al., 2005; Joseph and Plano, 2013; Lee et al., 2014). Upon encountering inducing cues the YscF needle may alter conformation, opening the channel to release YopN (Day et al., 2003) that then permits the secretion of other Yop substrates. The TyeA binding site on YopN is thought to encompass the C-terminal residues 248–293 (Iriarte et al., 1998; Cheng et al., 2001), as well as a secondary region involving residues 212–222 (Schubot et al., 2005). Hence, the deregulation of Yop synthesis observed in our strains with mutated *yopN* alleles could be explained by loss of YopN-TyeA binding.

**Figure 5:**
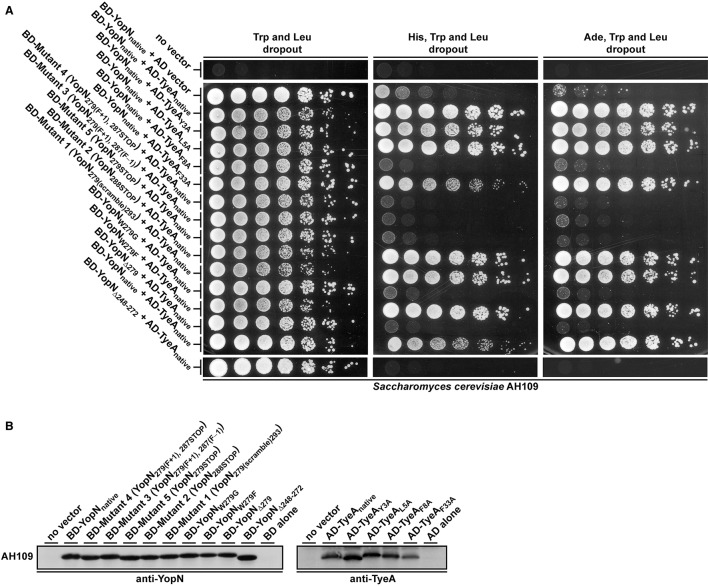
**Interaction analysis of YopN and TyeA fusions used in the Y2H assay**. All YopN variants were expressed fused to the GAL4 binding domain plasmid pGBKT7, while all TyeA variants were expressed fused to the GAL4 activation domain plasmid pGADT7 **(A)**. Containing *HIS3* and *ADE2* as reporter genes, the yeast strain *S. cerevisiae* AH109 was used as host for the two hybrid assay. Plasmid pairs were maintained in this strain via growth on dropout media lacking tryptophan (Trp) and leucine (Leu). Activation of the *HIS3* reporter was measured by yeast growth on this media also lacking histidine (His), whereas independent activation of the *ADE2* reporter was measured by yeast growth on equivalent media also lacking adenine (Ade). The extent of growth was recorded after 4 days following the plating of 2-fold serial dilutions of logarithmic phase yeast cultures. Results reflect trends in growth from three independent experiments in which different transformants were tested on each separate occasion. Yeast containing no vector or only YopN or TyeA, routinely failed to grow on the assay medium. To examine for stable expression of each variant in AH109, protein extracts were generated from yeast and separated by SDS-PAGE **(B)**. The YopN and TyeA fusions were identified by immunoblot analysis using anti-YopN and anti-TyeA antibody, respectively. In each case, a protein extract from AH109 harboring the relevant vector alone was included as a negative control. The approximate molecular weight of the BD::YopN variants were predicted to be around 48.0 kDa and the AD::TyeA variants around 22.0 kDa.

Consequently, we used the yeast two-hybrid system to investigate YopN-TyeA complex formation. Native *yopN* and manipulated alleles were translationally fused to the C-terminus of the Gal4 transcriptional activator DNA binding domain (BD) in pGBKT7, whereas the native *tyeA* allele was fused to the Gal4 activation domain in pGADT7. As indicated by yeast growth on selective media lacking either histidine or adenine, a strong interaction between native YopN and native TyeA corroborated previous studies (Figure 5A; Iriarte et al., 1998; Cheng et al., 2001; Schubot et al., 2005). In contrast, all three variants YopN_279(F+1), 287(F−1)_, YopN_279(F+1), 287STOP_, and YopN_279STOP_ completely lost an ability to engage with TyeA (Figure 5A, Mutants 3–5). This was similar to the lost TyeA binding by a YopN variant having a deletion of residues 248–272 encoding a coiled-coil domain that serves as an established TyeA anchor point (Figure 5A; Iriarte et al., 1998; Cheng et al., 2001; Schubot et al., 2005). Importantly, disruption of binding was not due to protein instability because these Gal4 BD fusions accumulated to levels in yeast that were comparable to the fusion made with native YopN (Figure 5B, Mutants 3–5). We also noted that even though the N-terminus of TyeA is the region that engages with YopN (Schubot et al., 2005), the AD-TyeA fusion that appends an additional domain at this position did not perturb the interaction. We also verified this interaction using the independent bacterial adenylate cyclase two-hybrid (BACTH) system. In this case, the T18 domain was appended to the YopN N-terminus and the T25 domain appended to the TyeA C-terminus (i.e., leaving a free YopN C-terminus to interact with a free TyeA N-terminus). Critically, the truncated YopN_Δ248−272_ deletion and all three YopN_279(F+1), 287(F−1)_, YopN_279(F+1), 287STOP_, and YopN_279STOP_ variants were once again unable to engage with TyeA, while a robust interaction between the two wild type proteins was readily apparent (electronic Supplementary Material, Figures S3A,C). Based on this information, we conclude that in Mutants 3–5 producing the YopN_279(F+1), 287(F−1)_, YopN_279(F+1), 287STOP_, and YopN_279STOP_ variants respectively, the YopN-TyeA regulatory complex is disrupted and this causes the deregulation of Yops synthesis and secretion, which in turn compromises T3S activity against immune cells.

### A new hydrophobic contact that supports YopN binding to TyeA

**Figure 6:**
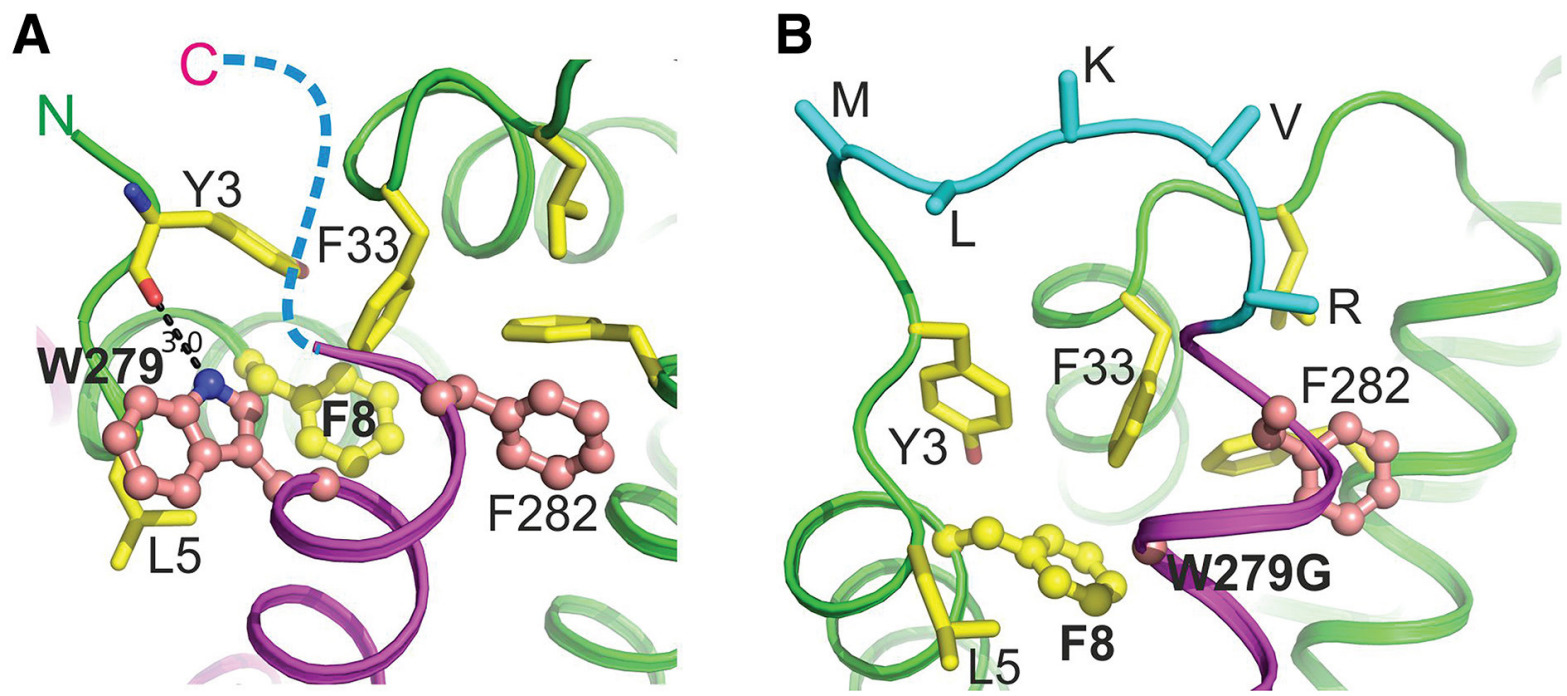
**Predicted molecular interactions between the C-terminus of YopN and the N-terminus of TyeA in the YopN-TyeA complex and YopN-TyeA fusion protein**. Cartoon diagram of a fragment of the crystal structure of the YopN-TyeA complex (RCSB PDB accession code 1XL3; **A**). The C-terminal helix of YopN is painted in magenta and TyeA is shown in green. Two C-terminal residues of YopN, significantly contributing to the binding interface, Trp279 and Phe282, are shown as balls-on-sticks with carbon and nitrogen atoms painted in pink and blue, respectively. Each of these two residues contributes about 10% to the total interactive area (1099 Å^2^), establishing hydrophobic interactions with TyeA. In addition, the nitrogen atom of the side chain of Trp279 forms a hydrogen bond with the main chain carbonyl group of Tyr3 in TyeA. Residues that interact with Trp279 and Phe282 are shown in sticks or balls-on-sticks (Phe8) with carbon, nitrogen, and oxygen atoms painted in yellow, blue, and red, respectively. The hydrogen bond between Trp279 and Tyr3 is shown with a dashed line (length 3.0 Å). Our study demonstrates the pivotal role of Trp279 of YopN and Phe8 of TyeA in the YopN-TyeA binding. The ten-residue C-terminus of YopN is unstructured (indicated by a blue dashed line) and, as we show here, plays no role in the binding. Cartoon diagram of a model of the YopN-TyeA fusion protein as a consequence of a mutated *yopN* allele containing an engineered *in cis* +1 frameshift mutation immediately downstream of codon 278 (Amer et al., 2013; **B**). The model was produced based on the crystal structure of the YopN-TyeA complex using program O. The connecting loop (cyan) was designed based on the search of loop library, keeping high restrains for stereochemistry. The side chains of residues at the C-terminus that are altered due to the +1 frame-shift were modeled using the most frequently found rotamer conformations. Only Cα and Cβ atoms are shown for the connecting loop residues. The interactive residues are shown as in **(A)**. The figure was generated by PYMOL (http://www.pymol.org/).

We wanted to explore why these three mutated *yopN* alleles produced a YopN product incapable of engaging with TyeA. A three dimensional structure of the YopN-TyeA complex has been reported (Schubot et al., 2005). The majority of the intermolecular interface sites concern hydrophobic contacts between the YopN residues W_216_, Y_213_, I_212_, V_271,_ and F_278_ with the respective partner residues S_6_, G_10_, V_13_, F_55,_ and M_51_ from TyeA (Schubot et al., 2005; Joseph and Plano, 2007). Thus, the region of YopN encompassing residues 279–287 may represent an extension of the TyeA contact site. To investigate this, we analyzed the available crystal structure of the YopN C-terminus in complex with TyeA and observed two additional C-terminal YopN residues W_279_ and F_292_ that seemingly form extensive hydrophobic interactions with TyeA and may thus contribute significantly to the binding energy (Figure 6A). The importance of these two residues were overlooked in the initial report (Schubot et al., 2005). Residue F_292_ remains unchanged following an engineered frame-shift (Amer et al., 2013) and in the mutants YopN_279(F+1), 287STOP_ and YopN_279(F+1), 287(F−1)_ generated herein (see Table 1), and therefore cannot be responsible for failed TyeA binding. However, in these variants the residue W_279_ is consistently exchanged for a G. Hence, we predicted that this W_279_ residue would be critical for YopN binding to TyeA. To test this, two *yopN* alleles were established that had only the single W_279_G mutation or a Δ279W mutation in which the entire codon was deleted. In parallel, an additional mutated *yopN* allele was created were W was exchanged for F (W_279_F) that possessed similar side chain properties. All three YopN variants were then assessed in parallel Y2H assays (Figure 5A) and BACTH assays (electronic Supplementary Material, Figure S3B) for their ability to bind TyeA. Data from both Y2H and BACTH analysis consistently revealed that YopN_*W*279*G*_ and YopN_Δ279W_ had completely lost the ability to engage TyeA, whereas the conservative mutant variant of YopN_*W*279F_ maintained robust TyeA binding akin to native YopN. Once again, this phenotype was not due to poor or unstable fusion expression in either assay host (Figure 5B and electronic Supplementary Material, Figure S3C). Thus, we have identified the residue W_279_ as a dominant hydrophobic contact point that contributes to stabilizing YopN-TyeA interactions.

### Identifying TyeA residues that reciprocate contacts with the YopN C-terminus

The TyeA residues S_6_, G_10_, V_13_, F_55,_ and M_51_ had previously been identified as contact points for YopN (Joseph and Plano, 2007). Our own analysis of the YopN-TyeA structure showed that the residues Y_3_, L_5_, F_8_ and F_33_ were also potential hydrophobic contact points on TyeA (Figure 6A). To study the importance of these interactions, all four TyeA residues were mutated to alanine, and then assessed for YopN binding in both Y2H assay (Figure 5A) and BACTH assay (electronic Supplementary Material, Figure S3D). Both assays consistently revealed that TyeA residue F_8_ was needed for interfacing with YopN. Importantly, at least for the Y2H assay we could confirm that the failure to detect an interaction was not due to poor protein production or unstable protein (Figure 5B), although this was not true for the BACTH assay where detection of these proteins was not possible (electronic Supplementary Material, Figure S3B). On the other hand, Y_3_, L_5,_ and F_33_ seemed not to be required, although again we could not confirm production of the F_33_ fusion in the BACTH assay (electronic Supplementary Material, Figure S3B), but all three were detected in the Y2H assay (Figure 5B).

**Figure 7:**
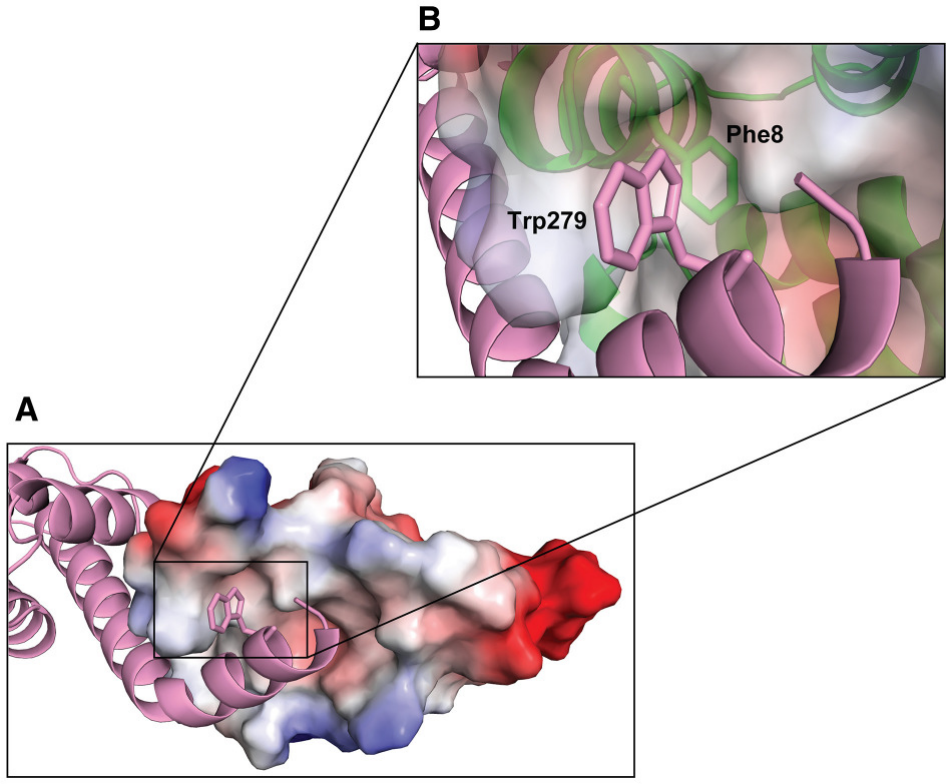
**Hydrophobic interaction between the C-terminus of YopN and TyeA**. TyeA is shown by electrostatic surface potential representation with negatively (red) and positively (blue) charged residues **(A)**. The C-terminal chain of YopN (magenta) is shown by ribbon view with Trp279 locating in the TyeA hydrophobic pocket. The insert **(B)** is a close up view of the TyeA hydrophobic pocket highlighting the close proximity between TyeA_(Phe8)_ and YopN_(Trp279)_.

This interaction data suggests that TyeA_*F*8_ makes direct contact with YopN_*W*279_ and the resultant hydrophobic contact contributes to stable YopN-TyeA complex formation. However, attempts to verify this using a cysteine crosslinking experiment on protein lysate from *Y. pseudotuberculosis* design to co-produce the engineered variants YopN_*W*279*C*_ and TyeA_*F*8*C*_ were inconclusive (data not shown). As a consequence, we examined closely the molecular surface of TyeA using available structural data. This revealed a definitive hydrophobic pocket that housed the F_8_ residue, and into which clearly projected the W_279_ side chain of YopN (Figure 7). Hence, TyeA_*F*8_ and YopN_*W*279_ are in close proximity where they most likely make direct and specific contact. Interestingly, both residues are a part of a large cluster of aromatic side chains that includes Y_3_, F_8_, F_33,_ and F_44_ in TyeA and W_279_ and F_282_ in YopN. These residues form nearly optimal T-shaped conformations, suggesting an important contribution of pi stacking interactions in this structure (Figure 6A). Hence, our data suggests that F_8_ and W_279_ are particularly important for stabilizing the YopN-TyeA complex by engaging in both hydrophobic and pi stacking interactions.

### The YopN_W279_-TyeA_F8_ hydrophobic contact is necessary for controlled T3SS activity

On the basis of their role in establishing a hydrophobic contact between YopN and TyeA, we would predict that their respective residues W_279_ and F_8_ are critical for T3SS activity. To test this we generated *in cis* mutations in *Y. pseudotuberculosis* to enable production of YopN_W279G_ and TyeA_F8A_, respectively. As controls, we also generated a further three isogeneic *in cis* mutations in *Y. pseudotuberculosis* to produce the TyeA_Y3A_, TyeA_L5A_, and TyeA_F33A_ variants, respectively. All five mutants were then compared to parental bacteria in a range of tests for T3SS activity, and the results are summarized in Table 1.

**Figure 8:**
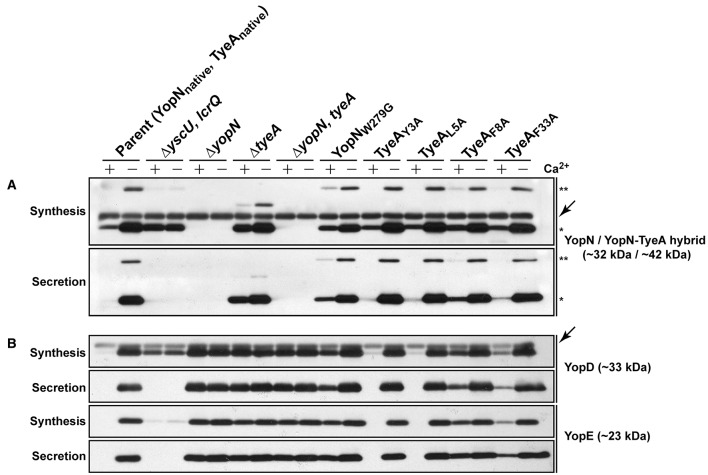
**The YopN_**W279**_-TyeA_**F8**_ contact is required for controlled Yop synthesis and secretion by ***in vitro*** grown ***Yersinia*****. Bacteria were grown in BHI medium either with (+) or without (−) Ca^2+^. Collected samples consisted of a mix of proteins contained within intact bacteria and associated with the outer bacterial surface that were retained in the bacterial pellet (Synthesis) or Yop proteins secreted free into the extracellular medium obtained from the cleared culture supernatants (Secretion). These were fractionated on a long 12% SDS-PAGE, wet-blotted onto PDVF membrane and then analyzed by immunoblot using polyclonal rabbit anti-YopN antiserum **(A)** or polyclonal rabbit anti-YopD and anti-YopE antiserum **(B)**. The single asterisk (^*^) highlights the singular YopN (~32 kDa) polypeptide, while the double asterisk (^**^) reveals the naturally produced and secreted ~42 kDa YopN-TyeA hybrid. Arrows (←) indicate non-specific protein bands recognized by the anti-YopN antiserum and the anti-YopD antiserum. The band appearing just above the nonspecific band in the Δ*tyeA* strain likely represents a frameshifting event that causes full-length YopN to be fused with the TyeA_Δ19−59_ deletion remnant resulting in a hybrid product that has a predicted molecular weight of ~38 kDa. Strains: Parent (YopN_native_, TyeA_native_), YPIII/pIB102; Δ*yscU, lcrQ* double mutant, YPIII/pIB75-26; Δ*yopN* null mutant, YPIII/pIB82; Δ*tyeA* null mutant, YPIII/pIB801a; Δ*yopN, tyeA* double mutant, YPIII/pIB8201a; YopN_*W*279*G*_, YPIII/pIB8223; TyeA_*Y*3*A*_, YPIII/pIB8221; TyeA_*L*5*A*_, YPIII/pIB8222; TyeA_*F*8*A*_, YPIII/pIB8220; TyeA_*F*33*A*_, YPIII/pIB8219. The theoretical molecular masses predicted from amino acid sequence are given in parentheses.

When examined for their ability to assemble YscF-needles at the bacterial surface and to control the production and secretion of components associated with the Ysc-Yop T3SS. It was evident that bacteria producing the TyeA_Y3A_ and TyeA_L5A_ variants maintain tight control of both YscF assembly (electronic Supplementary Material, Figure S2B), as well as Yops synthesis and secretion (Figure 8), to an extent that mirrored parental bacteria. Even bacteria producing TyeA_F33A_ displayed a near typical calcium dependent Yops synthesis and secretion profile, although a slight depression was observed during bacterial growth in the presence of calcium (Figure 8), and this corroborates elevated levels of surface-located YscF (electronic Supplementary Material, Figure S2B). Clearly however, surface assembled YscF (electronic Supplementary Material, Figure S2B) as well as Yops synthesis and secretion was deregulated in bacteria producing YopN_W279G_ or TyeA_F8A_ (Figure 8), and this was almost to the level observed for *Yersinia* bacteria harboring full length deletions of *yopN* or *tyeA*. These data were corroborated by the growth phenotype of these strains. The regulatory proficient TyeA_Y3A_- and TyeA_L5A_-producing bacteria were unable to grow without the addition of Ca^2+^, and this calcium dependent growth phenotype is shared by parental *Yersinia* (electronic Supplementary Material, Figure S1). In contrast, the regulatory deficient YopN_W279G_- and TyeA_F8A_-producing bacteria were unable to grow at 37°C irrespective of calcium, and this temperature sensitive growth phenotype is shared by *Yersinia* null mutants lacking *yopN* and/or *tyeA* (electronic Supplementary Material, Figure S1). Moreover, TyeA_F33A_-producing bacteria were modestly impaired in calcium dependent growth (electronic Supplementary Material, Figure S1), and this intermediate calcium dependent-like growth phenotype is consistent with elevated levels of surface located YscF and the slight defect observed in regulatory control of Yops synthesis and secretion.

To determine if compromised control of T3SS assembly and activity *in vitro* was an indicator of impaired *in vivo* function, we assessed bacterial viability in the presence of immune cells. After 6 h post-infection, it was evident that the mutants with most pronounced deregulation of T3SS activity—i.e., bacteria producing producing YopN_*W*279*G*_ or TyeA_*F*8*A*_ and to a lesser extent TyeA_*F*33*A*_, were all defective in their ability to resist immune cell killing (Figure 3B). Taken altogether, we suggest that at least residues F_8_ of TyeA and W_279_ of YopN promote fully controlled T3SS activity because they make a hydrophobic contact essential for stabilizing a YopN-TyeA interaction. Consistent with this finding is that alteration of these residues impact on the structural integrity of the two proteins as measured by YopN_*W*279*G*_ (Figure 4B) and TyeA_*F*8*A*_ (Figure 4C) being more prone to proteolytic digestion by endogenous proteases.

### C-terminal YopN contains functionally redundant sequence

We also examined the six residue coding sequence in the extreme C-terminus of YopN that overlaps by six codons with the N-terminal coding region of downstream *tyeA*. The generated Mutant 1 and Mutant 2 that produced YopN_288(scramble)293_ and YopN_288STOP_ respectively, both maintained appropriate control of T3S synthesis and secretion (Figure 2, Mutants 1 and 2) and protected bacteria from immune cell engulfment and intracellular killing by various antimicrobial processes as efficiently as bacteria harboring the native *yopN* allele, although the latter mutant was slightly impaired (Figure 3, Mutants 1 and 2). As these two mutant strains were also capable of a normal growth profile (electronic Supplementary Material, Figure S1, Mutants 1 and 2), we deliberately tested their ability to maintain T3SS activity during competitive infections of mice. Bacteria producing YopN_288STOP_ had a CI value of 0.33 and bacteria producing YopN_288(scramble)293_ had a CI value of 0.61 that was statistically not different from the CI value of 0.83 recorded for parental bacteria (electronic Supplementary Material, Table S1, *P* = 0.2222 and *P* = 0.5476, respectively). Thus, from these data it is apparent that both Mutant 1 and Mutant 2 producing YopN_288(scramble)293_ and YopN_288STOP_ respectively, still permit full T3S activity, even when forced to compete with parental bacteria for limiting nutrients and colonization niches, while simultaneously thwarting a directed and varied attack from the host immune response. These conclusions are reinforced by the fact that both the YopN_288(scramble)293_ and YopN_288STOP_ variants maintained robust binding to TyeA in both the Y2H assay (Figure 5A, Mutants 1 and 2) and the BACTH assay (electronic Supplementary Material, Figure S3B, Mutants 1 and 2).

### Disruption of YopN-TyeA hybrid formation

Despite the fact that the YopN C-terminus contains functionally redundant sequence, we considered the possibility that these six terminal residues that overlap with N-terminal TyeA sequence might be relevant in the context of YopN and TyeA being synthesized as a singular YopN-TyeA polypeptide in both *Y. pestis* and *Y. pseudotuberculosis* (Ferracci et al., 2004; Amer et al., 2013). As homologs of YopN and TyeA are commonly made as a singular polypeptide in other bacteria (Pallen et al., 2005a), it is possible that YopN-TyeA hybrid formation is functionally relevant under certain conditions. The structural consequence of this +1 frameshift has been modeled in Figure 6B. The altered C-terminal YopN sequence can act as a linker that maintains both YopN and TyeA structural integrity in the hybrid fusion that compensates for loosing pivotal hydrophobic contacts necessary for complex formation of the singularly produced polypeptides (e.g., between YopN_W279_ and TyeA_F8_). Hence, we inspected YopN-TyeA hybrid formation in our six C-terminal mutated YopN mutants after growth in BHI broth restrictive (plus Ca^2+^) and permissive (minus Ca^2+^) for T3S. Bacteria producing YopN_288(scramble)293_ (Figure 2A) or YopN_W279G_ (Figure 8A) formed a natural chimera with TyeA to similar levels as produced by parental bacteria. However, relative to the single YopN polypeptide the level of hybrid production was severely reduced in bacteria producing the four other YopN mutants (Figure 2A). In fact, hybrid formation with YopN_279(F+1), 287(F−1)_ was undetected (Figure 2A). Thus, it is possible to manipulate YopN amounts produced alone relative to when produced as a YopN-TyeA hybrid fusion, and the latter appears to be influenced by the six codon overlap between the end of YopN and the beginning of TyeA.

## Discussion

We have performed a functional characterization of the YopN C-terminus. This revealed a segment encompassing residues 279–287 that performs important functions in the control of T3S activity. Likely this occurs through the positioning of the residue W_279_ to facilitate hydrophobic intermolecular contact with the F_8_ residue of TyeA and stabilization of an aromatic cluster at the TyeA-YopN interface. The consequence of these interactions is to contribute to the formation of a functional YopN conformation. On the other hand, YopN has evolved with six terminal residues (288–293) that serve no obvious function. However, we speculate that this strategically situates the end of *yopN* in overlap with the start of *tyeA*, which may aid in controlling a programmed +1 frameshifting event that serves to join YopN with TyeA to form a larger chimeric protein and also control the production of singular TyeA.

Mutants 3–5 that altered YopN sequence between residues 279–287 (i.e., generating the YopN_279(F+1), 287(F−1)_, YopN_279(F+1), 287STOP_, and YopN_279STOP_ variants respectively) resulted in bacteria with dysfunctional T3SSs, as measured by both *in vitro* and *in vivo* tests. The variants YopN_279(F+1), 287STOP_ and YopN_279STOP_ did not display any increase in *in vivo* susceptibility to proteolysis, indicating that their defective phenotypes are caused more likely by a defect in YopN function *per se*, rather than by disrupting the structural integrity of YopN folding. However, the variant YopN_279(F+1), 287(F−1)_ did displayed some reduction in stable protein levels when compared to native YopN. Hence, the introduced mutations have probably brought about some modest structural change, or even altered the ability to bind target proteins, which in turn has heighten its sensitivity to proteolysis. On this note, it is interesting that in bacteria lacking the YopN anchor, TyeA, native YopN was considerably more unstable then any of our engineered mutants. This cannot be due to low levels of YopN production—perhaps by residual YopN plugging the secretion channel to cause feedback inhibition of Yop synthesis—because this *tyeA* mutant is quite obviously de-regulated for Yops production and secretion (this study; Amer et al., 2013). Rather, it suggests that TyeA targets YopN, and this interaction stabilizes YopN cytoplasmic pools. This stabilizing effect of TyeA must function conjointly with the T3S SycN-YscB cochaperone, which is a known stabilizer and secretion pilot of YopN (Day and Plano, 1998; Cheng et al., 2001; Day et al., 2003). Thus, TyeA would serve at least two functions in complex with YopN—the first to stabilise YopN and the second to anchor YopN as it plugs the secretion channel. Thus, an inability to bind TyeA renders the YopN_279(F+1), 287STOP_, YopN_279(F+1), 287(F−1)_, and YopN_279STOP_ variants incapable of plugging the T3S channel, thus surrendering any possibility to impart meticulous environmental control of T3S in *Yersinia*.

A previous study had identified the YopN residues W_216_, Y_213_, I_212_, V_271_, and F_278_ as being critical for engaging with TyeA (Schubot et al., 2005). In one other study, the TyeA residues S_6_, G_10_, V_13_, F_55_, and M_51_ were revealed to be important for YopN binding (Joseph and Plano, 2007). Herein, we have combined analyses of available structural data with various protein-protein interaction assays to identify a specific hydrophobic contact between YopN_W279_ and TyeA_F8_. So important is this interaction to YopN function that alteration of either residue severely disrupts T3SS activity by *Y. pseudotuberculosis*. Interestingly, a *BLASTP* analysis of all known YopN amino acid sequences revealed a prominent foci of sequence diversity in the C-terminus that also incorporates the TyeA binding domain between residues 248 and 272 (data not shown; Iriarte et al., 1998; Cheng et al., 2001; Schubot et al., 2005). Yet a similar analysis of TyeA revealed it to be generally well conserved across all pathogenic *Yersinia* isolates (data not shown). Hence, we speculate that this YopN C-terminal region may have evolved specific sequence variations as a means to strategically modulate TyeA binding avidity to customize the extent of Ysc-Yop T3S control imparted by the YopN-TyeA complex in the different pathogenic variants of human pathogenic *Yersinia*. We are currently testing this hypothesis experimentally, with the idea that this type of fine-tuning of T3S control may afford certain *Yersinia* isolates the potential to facilitate unique niche adaptations.

On the other hand, the extreme terminal six residues of YopN appeared to serve no obvious purpose in the control and/or activity of the Ysc-Yop T3SS of *Y. pseudotuberculosis*, at least under the *in vitro* and *in vivo* experimental conditions tested herein. These data corroborate studies that have appended fusions to the C-terminus of YopN without loss of function (Day et al., 2003; Garcia et al., 2006). Yet this region strategically overlaps with the N-terminus of TyeA, such that upon a +1 frameshifting event can produce a YopN-TyeA hybrid (Ferracci et al., 2004). Engineered mutants of *Y. pseudotuberculosis* designed to mimic this endogenous +1 frameshift to produce only the YopN-TyeA hybrid have been examined (Amer et al., 2013). These mutants maintained *in vitro* low Ca2+-dependent control of substrate T3S, although they were unable to control polarized translocation of effectors into the cytosol of eukaryotic cells, which reduced their ability to survive *in vivo* infections of mice (Amer et al., 2013). Hence, the formation of a YopN-TyeA hybrid in Yersinia can have functional consequences for T3SS activity. This corroborates other studies showing that programmed translational +1 frameshifting is a way to regulate the production or diversity of various protein entities (Farabaugh, 1996; Baranov et al., 2002; Namy et al., 2004; Buchan and Stansfield, 2007; Dinman, 2012). As nucleic acid architecture and environmental factors influence frameshifting events (Schwartz and Curran, 1997; Björk et al., 1999; Kontos et al., 2001; McNulty et al., 2003; Higashi et al., 2006; Hansen et al., 2007), the identification of such factors that modulate YopN-TyeA hybrid formation in Yersinia would have biological relevance.

Our data herein suggests two architectural features that potentially influence hybrid formation. The first is the six codon overlap between the end of YopN and the beginning of TyeA. Even though this region appears functionally redundant, the disparity in hybrid formation in Mutant 1 bacteria producing YopN288(scramble)293 compared to Mutant 2 and Mutant 4 bacteria producing YopN288STOP and YopN279(F+1), 287STOP respectively, suggests that this region obviously has cause to affect YopN and TyeA production as singular entities and as a fused unit. The second feature concerns the position of the tyeA Shine-Dalgarno (SD) sequence relative to the upstream potential +1 frameshifting site (codons 278 and 279 of yopN), the downstream tyeA initiation codon, and the downstream yopN termination codon. Particularly for the YopN288STOP variant, the tyeA initiation codon is displaced relative to a putative Shine-Dalgarno sequence such that a +1 frameshift may no longer give productive translation if the ribosome encounters a premature stop codon. This is relevant given how the SD location relative to other architectural features of the coding sequence does affect +1 frameshifting frequency (Weiss et al., 1988; Chen et al., 1994; Li et al., 2012). Thus, a future goal of ours is to investigate whether the length and position of the tyeA SD sequence relative to the tyeA start and the yopN stop may have evolved to promote YopN-TyeA hybrid formation.

In summary, this study has identified a critical point of contact between YopN and TyeA that is necessary for ensuring the correct functional orientation of YopN. A YopN-TyeA hybrid is also produced possibly via a translational +1 frameshift after codon 278 of *yopN* (Ferracci et al., 2004; Amer et al., 2013). A YopN-TyeA hybrid produced by *Y. pseudotuberculosis* is stable, but does not retain full function *in vivo* (Amer et al., 2013). Structural modeling of this singular hybrid polypeptide indicated an altered conformation compared to the YopN-TyeA heterocomplex. Therefore, we believe that the YopN-TyeA heterocomplex has a defined conformation conferred by specific hydrophobic contacts, and this is critical for full YopN function, the importance of which we have demonstrated here.

## Author contributions

AA, JG, TC, and ÅF carried out the laboratory work. TC and AZ performed the structural modeling. AA, JG, and MF designed the experiments and wrote the manuscript; all authors helped draft the manuscript, and gave their final approval for publication.

### Conflict of interest statement

The authors declare that the research was conducted in the absence of any commercial or financial relationships that could be construed as a potential conflict of interest.
