# Supplementary material for: YopN and TyeA Hydrophobic Contacts Required for Regulating Ysc-Yop Type III Secretion Activity by Yersinia pseudotuberculosis
Source: Front Cell Infect Microbiol. 2016 Jun 21;6:66. doi: 10.3389/fcimb.2016.00066 (PMC4914553; doi:10.3389/fcimb.2016.00066)
Supplement: Supplementary file 1 [file DataSheet1.DOC]

**YopN and TyeA hydrophobic contacts required for regulating Ysc-Yop type III secretion activity by *Yersinia pseudotuberculosis***

Ayad A. A. Amer,^1,2,6,§^ Jyoti M. Gurung,^1,2,§^ Tiago R. D. Costa,^1,2,7^ Anton V. Zavialov,^4,5^ Åke Forsberg,^1,2,3^ and Matthew S. Francis^1,2^

^1^Department of Molecular Biology, Umeå University, Umeå, Sweden

^2^Umeå Centre for Microbial Research (UCMR), Umeå University, Umeå, Sweden

^3^Laboratory for Molecular Infection Medicine Sweden (MIMS), Umeå University, Umeå, Sweden ^4^Department of Molecular Biology, Uppsala BioCenter, Swedish University of Agricultural Sciences, Uppsala, Sweden

^5^Department of Chemistry, University of Turku, Turku, Finland

**Electronic supplementary material**

**MATERIALS AND METHODS:**

**Mouse co-infections and competitive index measurements**

**Low-calcium growth measurement**

**Surface YscF polymerization**

**Plasmid construction, transformation and BACTH analysis**

**TABLES:**

**Table S1| Competitive index for mice colonization**

**Table S2| Strains and plasmids used in this study**

**Table S3| Oligonucleotides used in this study**

**FIGURES:**

**Figure S1| Low calcium dependent growth of *yopN* mutants**

**Figure S2| Chemically cross-linked surface-located YscF**

**Figure S3| Interaction analysis of YopN and TyeA fusions used in the BACTH assay**

**Materials and Methods**

**Mouse co-infections and competitive index measurements**

As described previously , a polar insertion of the gene encoding for a 349 amino acid inner membrane oligo-dipeptide/nickel ABC transporter permease (annotated as YPK_3687 in *Y. pseudotuberculosis* YPIII) was introduced into our mutants by a single cross-over of the pUA066 mutagenesis plasmid (Table 1). Connected to creating a polar mutation in YPK_3687, integration of the mutagenesis plasmid conferred to these bacteria a Cm^R^ marker for counter-selection against Cm^S^ parental bacteria. In our standard *in vitro* LCR growth assays and Ysc-Yop synthesis and secretion assays, no difference between isogenic single (Cm^S^) and double (Cm^R^) mutants was observed.

Female eight-week-old BALB/c mice (Taconic, Denmark) were given food and water *ad libitum*. Then groups of five mice were deprived of food and water 16 h prior to oral infection. For infection, bacteria were grown overnight in 50 ml LB broth at 26°C, then pelleted and serially diluted in sterile tap water supplemented with 150 mM NaCl. Serial dilutions were plated to record CFU/ml and their corresponding A_600_ measured to establish the volume of culture needed to inoculate 50 ml of sterile drinking water with 2.5 x 10^9^ viable mutant bacterial cells (Cm^R^) and 2.5 x 10^9^ viable parental bacterial cells (Cm^S^). Mice were allowed to drink from this inoculated water for 6 hours. Measurement of CFU was again performed to calculate the input ratio of Cm^R^ bacteria compared to the total inoculated dose. At day 4 post infection, spleens were harvested aseptically in sterile PBS, homogenized, and plated for bacterial CFU analysis to obtain an output ratio of Cm^R^ bacteria among the total viable population. In turn, the competitive index was determined as the ratio of percent Cm^R^ output versus percent Cm^R^ input.

**Low-calcium growth measurements**

As described previously , *Yersinia* low-calcium-response growth phenotypes were measured by monitoring absorbance at 600 nm during bacterial growth under high- and low-Ca^2+^ conditions at 37°C in liquid Thoroughly Modified Higuchi’s (TMH) medium (minus Ca^2+^) or TMH medium supplemented with 2.5 mM CaCl_2_ (plus Ca^2+^). As controls, we monitored the calcium dependent (CD) growth of parental *Yersinia* (YPIII/pIB102) bacteria that are unable to grow in the absence of Ca^2+^ at 37°C, and the temperature sensitive (TS) growth of *Yersinia* bacteria lacking both the yopN and *tyeA* alleles (YPIII/pIB8201a), since these bacteria cannot grow at 37°C .

**Surface YscF polymerization**

Overnight cultures from *Yersinia* strains were grown with shaking at 26°C in 2 ml of BHI broth supplemented with 2.5mM CaCl_2_. Subsequently, 0.1 volumes of bacterial suspension were sub-cultured into 3 ml fresh media and incubated for 3 hour at 37°C. After the each culture was standardized by A_600_, 1 ml volumes were harvested by centrifugation at 8000 *g* for 5 min at 4°C. Each bacterial pellet was gently resuspended in 1 ml of cold 20 mM HEPES, 2.5 mM CaCl_2_ (pH 8). Bacterial surface proteins were cross-linked for 30 min at RT with the non-cleavable, membrane-impermeable, amine-reactive cross-linker Pierce BS^3^ (Thermo Scientific) at a final concentration of 5 mM. Cross-linking reactions were quenched for 15 min by addition of Tris-HCl (pH 8.0) to a final concentration of 20 mM. Cell fractions were collected by centrifugation at 12200 *g* for 5 min at 4°C. Bacterial pellets were then resuspended in 100 µl of 1X sample buffer and analyzed by 18 % acrylamide SDS PAGE and immunoblotting with rabbit anti-YscF polyclonal antiserum (a gift from Hans Wolf-Watz) that underwent several rounds of immunoadsorption with purified YscF to enhance its monospecificity.

**Plasmid construction, transformation and BACTH analysis**

The various *tyeA* and *yopN* alleles used in this study were cloned as a translational fusion into the XbaI/EcoRI site of the pKT25, pKNT25, pUT18, or pUT18C vectors for BACTH analysis (Euromedex, Souffelweyersheim, France) (Table 1). Pairs of BACTH vectors expressing CyaA T18 and T25 fusions to ‘bait’ and ‘prey’ proteins were co-transformed into chemically competent E. coli BTH101 and selected at 37°C by overnight growth on LB agar containing the appropriate antibiotics, 0.5 mM isopropyl-β-D-thiogalactopyranoside (IPTG) and 40 μg/ml 5-bromo-4-chloro-3-indolyl-β-D-galactopyranoside (X-Gal). Well isolated single colonies were then selected for protein-protein interaction analysis as previously described , with the exception that bacterial lysates were prepared from 1 ml of culture with the addition of 50 µl 0.1% SDS and 100 µl Chloroform. β-galactosidase activity was represented in Units/mg dry weight bacteria according to the manufacturer’s direction (Euromedex). Data are a representative of at least four independent experiments performed in triplicate. A positive interaction was considered only if the β-galactosidase activity level was at least three-fold higher than that measured for the negative control plasmids (expressing T18 and/or T25 alone).

**Table S1|** Competitive index for mice colonization

| **Strain (Cm^R^)^a^** | **Input CFU/mouse^b^** | **Output CFU/ml^c^** | **Competitive index^d^** | **Fold difference^e^** |
| --- | --- | --- | --- | --- |
| Parental (YopN_wt_) | 3.49 × 10^8^ (59.6%) | 1) 1.65 × 10^6^ (88.5%) 2) 5.97 × 10^5^ (22.9%) 3) 4.12 × 10^6^ (9.7%) 4) 9.43 × 10^5^ (85.0%) 5) 1.24 × 10^5^ (62.6%) | 0.83 ± 0.28 | na |
| Δ*yopN, tyeA* | 2.34 × 10^8^ (39.6%) | 1) 0 2) 1.0 × 10^1^ (0.006%) 3) 0 4) 0 5) 8.0 × 10^1^ (0.012%) | 0.00007 ± 0.00005 (**, *P*=0.0079) | 11857 |
| YopN_288(scramble)293_ | 2.17 × 10^8^ (34.5%) | 1) 6.36 × 10^4^ (68.7%) 2) 7.84 × 10^3^ (4.6%) 3) 2.41 × 10^4^ (3.6%) 4) 1.18 × 10^5^ (16.9%) 5) 1.61 × 10^5^ (12.4%) | 0.61 ± 0.35 (ns, *P*=0.5476) | 1.36 |
| YopN_288STOP_ | 2.80 × 10^8^ (42.3%) | 1) 5.66 × 10^6^ (3.3%) 2) 1.07 × 10^3^ (28.2%) 3) 6.56 × 10^5^ (16.5%) 4) 9.62 × 10^4^ (18.4%) 5) 4.21 × 10^4^ (4.0%) | 0.33 ± 0.11 (ns, *P*=0.2222) | 6.63 |

^a^ All mutant strains were selected for on the basis of a Cm^R^ marker incorporated via a polar mutation introduced *in cis* in the chromosome locus YPK_3687 through a single cross-in of the mutagenesis plasmid pUA066 .

^b^ Calculated from the amount of water consumed per cage and then averaged over 5 mice. In parentheses are percentages that correspond to the proportion of total bacteria (*i.e.*: Cm^S^ + Cm^R^) in the inoculation water that are Cm^R^.

^c^ Cm^R^ bacteria recovered from each individual spleen. In parentheses are percentages that correspond to the proportion of total bacteria (*i.e.*: Cm^S^ + Cm^R^) recovered from an individual spleen that are Cm^R^.

^d^ Competitive index (CI) is a ratio of the proportion of Cm^R^ CFU recovered from infected mice (**expressed as a** percentage of total CFU output – *see* footnote ‘c’, Table S2) and the proportion of Cm^R^ CFU inoculated into mice (**expressed as a** percentage of total CFU input – *see* footnote ‘b’) when in direct competition with the Cm^S^ parental strain that is wild type for the *yopN* allele. A value of 1.0 therefore indicates that both parent and mutant bacteria compete equally well in infecting, colonizing and spreading systemically in orally infected mice. On the other hand, values progressively falling below 1.0 are indicative of the degree of attenuation shown by the Cm^R^ mutant bacteria relative to the parent (*yopN*_wt_). Numbers are the mean ± standard error of independent CI values derived from five individual mice inoculated through contaminated drinking water. Data sets were analyzed using the non-parametric two-tailed Mann-Whitney U-test. Analysis was performed using GraphPad Prism version 5.00 for Windows. Only differences between mutants and parent with a p-value < 0.05 were considered significant. ns – no significant difference

^e^ Fold difference is the ratio between CI of parental (*yopN*_wt_) (**numerator**) and mutated derivatives of *yopN* (denominator).

**Table S2|** Strains and plasmids used in this study

| **Strains and plasmids** | **Relevant genotype or phenotype** | **Source or reference** |
| --- | --- | --- |
| Strain |  |  |
| *E. coli* | | |
| DH5 | F^−^, *recA*1, *endA*1, *hsdR*17, *supE*44, *thi*-1, *gyrA*96, *relA*1 | Vicky Shingler |
| S17-1λ*pir* | *recA*, *thi*, *pro*, *hsdR^-^M^+^,* Sm^R^, <RP4:2-Tc:Mu:Ku:Tn7>Tp^R^ |  |
| *Y. pseudotuberculosis* | | |
| YPIII/pIB102 | *yadA*::Tn5, Km^R^ (wild type) | Hans Wolf-Watz |
| YPIII/pIB75 | pIB102, *yscU* in frame deletion of codons 25-329, Km^R^ |  |
| YPIII/pIB75-26 | pIB102, *yscU* and *lcrQ* double mutant, Km^R^ | (2) |
| YPIII/pIB202 | pIB102, *yscF* in frame deletion of codons 11-69, Km^R^ | (8) |
| YPIII/pIB619 | pIB102, *yopB* and *yopD* full length deletion, Km^R^ | (9) |
| YPIII/pIB82 | pIB102, near full length deletion of *yopN*, Km^R^ |  |
| YPIII/pIB8203 | pIB102, *yopN* in frame deletion of codons 64-110, Km^R^ | This study |
| YPIII/pIB8202 | pIB102, *yopN* in frame deletion of codons 248-272, Km^R^ | This study |
| YPIII/pIB801a | pIB102, *tyeA* in frame deletion of codons 19-59, Km^R^ |  |
| YPIII/pIB8201a | pIB102, in frame double deletion of *yopN* and *tyeA*, Km^R^ |  |
| YPIII/pIB8212 | pIB102, *yopN* allele with a nonsense (‘TAG’) mutation inserted after codon 287 to give YopN_288STOP_, Km^R^ | This study |
| YPIII/pIB8213 | pIB102, full length *yopN* allele with semi-conservative substitutions in the codons at the extreme 3-prime end to give YopN_288(scramble)293_ but maintain native *tyeA* sequence, Km^R^ | This study |
| YPIII/pIB8207 | pIB102, truncated *yopN* allele with a +1 frameshift deletion mutation (‘T’) after codon 278 and a nonsense mutation (‘TAG’) inserted after new codon 286 to give YopN_279(F+1), 287STOP_, Km^R^ | This study |
| YPIII/pIB8208 | pIB102, full length *yopN* allele with a +1 frameshift deletion mutation (‘T’) after codon 278 and a compensatory insertion mutation (‘A’) immediately after new codon 287 to give YopN_279(F+1), 287(F−1)_, Km^R^ | This study |
| YPIII/pIB8209 | pIB102, *yopN* allele with a nonsense (‘TAG’) mutation inserted immediately after codon 278 to give YopN_279STOP_, Km^R^ | This study |
| YPIII170/pIB102 | *In cis* polar mutation of YPK_3687 in the parental background, Cm^R^, Km^R^ |  |
| YPIII170/pIB8201a | *In cis* polar mutation of YPK_3687 in the *yopN* and *tyeA* background, Cm^R^, Km^R^ |  |
| YPIII170/pIB8212 | *In cis* polar mutation of YPK_3687 in the YopN_288STOP_-producing background, Cm^R^, Km^R^ | This study |
| YPIII170/pIB8213 | *In cis* polar mutation of YPK_3687 in the YopN_288(scramble)293_-producing background, Cm^R^, Km^R^ | This study |
| YPIII/pIB8216 | pIB102, *yopN* allele with a missense mutation at codon 279 (Trp_TGG_🡪Phe_TTC_) to give a YopN_W279F_, Km^R^ |  |
| YPIII/pIB8218 | pIB102, *yopN* allele with a deletion of codon 279 to give a YopN_Δ279W_, Km^R^ |  |
| YPIII/pIB8219 | pIB102, *tyeA* allele with a missense mutation at codon 33 (Phe_TTT_→Ala_GCA_) to give a TyeA_F33A_, Km^R^ | This study |
| YPIII/pIB8220 | pIB102, *tyeA* allele with a missense mutation at codon 8 (Phe_TTT_→Ala_GCA_) to give a TyeA_F8A_, Km^R^ | This study |
| YPIII/pIB8221 | pIB102, *tyeA* allele with a missense mutation at codon 3 (Tyr_TAC_→Ala_GCA_) to give a TyeA_Y3A_, Km^R^ | This study |
| YPIII/pIB8222 | pIB102, *tyeA* allele with a missense mutation at codon 5 (Leu_CTT_→Ala_GCA_) to give a TyeA_L5A_, Km^R^ | This study |
| YPIII/pIB8223 | pIB102, *yopN* allele with a missense mutation at codon 279 (Trp_TGG_→Gly_GGC_) to give a YopN_W279G_, Km^R^ | This study |
| Plasmid | | |
| pKT25-Zip | Derivative of pKT25, having an leucine zipper motif of GCN4 fused in frame to the C-terminal end of CyaA_T25_, Km^R^ | Euromedex |
| pUT18C-Zip | Derivative of pUT18, having an leucine zipper motif of GCN4 fused in frame to the C-terminal end of CyaA_T18_, Ap^R^ | Euromedex |
| pKT25 | Cloning vector for creating in-frame fusions at the C-terminal end of CyaA_T25_, Km^R^ | Euromedex |
| pKNT25 | Cloning vector for creating in-frame fusions at the N-terminal end of CyaA_T25_, Km^R^ | Euromedex |
| pUT18 | Cloning vector for creating in-frame fusions at the N-terminal end of CyaA_T18_, Ap^R^ | Euromedex |
| pUT18C | Cloning vector for creating in-frame fusions at the C-terminal end of CyaA_T18_, Ap^R^ | Euromedex |
| pAA211 | pKNT25 with full length *tyeA* coding residues 1 to 92 (TyeA_1-92_-CyaA_T25_), Km^R^ | This study |
| pJMG131 | pKNT25 with full length *tyeA* coding residues 1 to 92 having the missense mutation Y_3_A (TyeA_Y3A_-CyaA_T25_), Km^R^ | This study |
| pJMG134 | pKNT25 with full length *tyeA* coding residues 1 to 92 having the missense mutation L_5_A (TyeA_L5A_-CyaA_T25_), Km^R^ | This study |
| pJMG132 | pKNT25 with full length *tyeA* coding residues 1 to 92 having the missense mutation F_8_A (TyeA_F8A_-CyaA_T25_), Km^R^ | This study |
| pJMG133 | pKNT25 with full length *tyeA* coding residues 1 to 92 having the missense mutation F_33_A (TyeA_F33A_-CyaA_T25_), Km^R^ | This study |
| pAA203 | pUT18C with full length *yopN* coding residues 1 to 293 (CyaA_T18_-YopN_1-293_), Ap^R^ | This study |
| pMF911 | pUT18C with truncated *yopN* coding residues 1 to 286 with an altered C-terminus (CyaA_T25_-YopN_279(F+1), 287STOP_), Ap^R^ | This study |
| pMF912 | pUT18C with full length *yopN* coding residues 1 to 293 with an altered C-terminus (CyaA_T18_-YopN_279(F+1), 287(F−1)_), Ap^R^ | This study |
| pMF913 | pUT18C with truncated *yopN* coding residues 1 to 278 (CyaA_T18_-YopN_279STOP_), Ap^R^ | This study |
| pMF914 | pUT18C with truncated *yopN* coding residues 1 to 287 (CyaA_T18_-YopN_288STOP_), Ap^R^ | This study |
| pMF915 | pUT18C with full length *yopN* coding residues 1 to 293 with a scrambled extreme C-terminus (CyaA_T18_-YopN_288(scramble)293_), Ap^R^ | This study |
| pJMG128 | pUT18C with full length *yopN* coding residues 1 to 293 and having the missense mutation W_279_G (CyaA_T18_-YopN_W279G_), Ap^R^ | This study |
| pJMG129 | pUT18C with full length *yopN* coding residues 1 to 293 and having the missense mutation W_279_F (CyaA_T18_-YopN_W279F_), Ap^R^ | This study |
| pJMG130 | pUT18C with near full length *yopN* having a deletion of codon 279 (CyaA_T18_-YopN_Δ279W_), Ap^R^ | This study |
| pMF917 | pUT18C with *yopN* coding a deletion of residues 248 to 272 (CyaA_T18_-YopN_Δ248-272_), Ap^R^ | This study |
| pGADT7 | *LEU2*, Amp^R^ | Clontech Laboratories |
| pAA200 | EcoRI/*Bam*HI PCR fragment of full length *yopN* and cloned in pGADT7, *LEU2,* Amp^R^ | This study |
| pAA206 | EcoRI/*Bam*HI PCR fragment of full-length *tyeA* in pGADT7, *LEU2,* Amp^R^ | This study |
| pJMG124 | EcoRI/*Bam*HI PCR fragment of full-length *tyeA* with the missense mutation Y_3_A and cloned in pGADT7, *LEU2,* Amp^R^ | This study |
| pJMG125 | EcoRI/*Bam*HI PCR fragment of full-length *tyeA* with the missense mutation L_5_A and cloned in pGADT7, *LEU2,* Amp^R^ | This study |
| pJMG126 | EcoRI/*Bam*HI PCR fragment of full-length *tyeA* with the missense mutation F_8_A and cloned in pGADT7, *LEU2,* Amp^R^ | This study |
| pJMG127 | EcoRI/*Bam*HI PCR fragment of full-length *tyeA* with the missense mutation F_33_A and cloned in pGADT7, *LEU2,* Amp^R^ | This study |
| pGBKT7 | *TRP1*, Km^R^ | Clontech Laboratories |
| pAA201 | *Eco*RI/*Bam*HI PCR fragment of full-length *yopN* and cloned in pGBKT7, *TRP1*, Km^R^ | This study |
| pJMG080 | EcoRI/*Bam*HI PCR fragment of truncated *yopN* coding residues 1 to 286 that has the 279_(F+1)_ insertion and 287_STOP_ nonsense mutations and cloned in pGBKT7, *TRP1*, Km^R^ | This study |
| pJMG081 | EcoRI/*Bam*HI PCR fragment of full length *yopN* with the 279_(F+1)_ insertion and 287_(F−1)_ deletion mutations and cloned in pGBKT7, *TRP1*, Km^R^ | This study |
| pJMG082 | EcoRI/*Bam*HI PCR fragment of truncated *yopN* coding residues 1 to 278 that has the 279_STOP_ nonsense mutation and cloned in pGBKT7, *TRP1*, Km^R^ | This study |
| pJMG083 | EcoRI/*Bam*HI PCR fragment of truncated *yopN* coding residues 1 to 287 that has the 288_STOP_ nonsense mutation and cloned in pGBKT7, *TRP1*, Km^R^ | This study |
| pJMG084 | EcoRI/*Bam*HI PCR fragment of full length *yopN* with the mutation 288_(scramble)_293 and cloned in pGBKT7, *TRP1*, Km^R^ | This study |
| pJMG121 | EcoRI/*Bam*HI PCR fragment of full length *yopN* with the missense mutation W_279_G and cloned in pGBKT7, *TRP1*, Km^R^ | This study |
| pJMG122 | EcoRI/*Bam*HI PCR fragment of full length *yopN* with the missense mutation W_279_F and cloned in pGBKT7, *TRP1*, Km^R^ | This study |
| pJMG123 | EcoRI/*Bam*HI PCR fragment of near full length *yopN* with a deletion of codon 279 and cloned in pGBKT7, *TRP1*, Km^R^ | This study |
| pJMG085 | EcoRI/*Bam*HI PCR fragment of truncated *yopN* with a deletion of Δ64-110 and cloned in pGBKT7, *TRP1*, Km^R^ | This study |
| pJMG086 | EcoRI/*Bam*HI PCR fragment of truncated *yopN* with a deletion of Δ248-272 and cloned in pGBKT7, *TRP1*, Km^R^ | This study |
| pAA207 | *Eco*RI/*Bam*HI PCR fragment of full-length *tyeA* and cloned in pGBKT7, *TRP1*, Km^R^ | This study |
| pUA066 | pNQ705-derived mutagenesis vector for the construction of a polar insertion in YPK_3687, Cm^R^ |  |
| pDM4 | Suicide vector with oriR6K, sacB, Cm^R^ | Debra Milton |
| pSF018 | *Sal*I/*Xba*I PCR fragment of *yopN* with a deletion of codons 64 to 110 in pDM4, Cm^R^ | This study |
| pSF020 | *Sal*I/*Xba*I PCR fragment of *yopN* with a deletion of codons 248 to 272 in pDM4, Cm^R^ | This study |
| pAA249 | *Sal*I/*Xba*I PCR fragment of *yopN* with a nonsense (‘TAG’) mutation inserted after codon 287 in pDM4, Cm^R^ | This study |
| pAA250 | *Sal*I/*Xba*I PCR fragment of *yopN* with semi-conservative substitutions in the codons at the extreme 3-prime end in pDM4, Cm^R^ | This study |
| pAA244 | *Sal*I/*Xba*I PCR fragment of *yopN* with a +1 frameshift deletion mutation (‘T’) after codon 278 and a nonsense mutation (‘TGA’) inserted after new codon 286 in pDM4, Cm^R^ | This study |
| pAA245 | *Sal*I/*Xba*I PCR fragment of *yopN* allele with a +1 frameshift deletion mutation (‘T’) after codon 278 and a compensatory insertion mutation (‘A’) immediately after new codon 287 in pDM4, Cm^R^ | This study |
| pAA246 | *Sal*I/*Xba*I PCR fragment of *yopN* with a nonsense (‘TAG’) mutation inserted after codon 278 in pDM4, Cm^R^ | This study |
| pJMG159 | *Sal*I/*Xba*I PCR fragment of *yopN* with a missense mutation at codon 279 (Trp_TGG_→Gly_GGC_) in pDM4, Cm^R^ | This study |
| pJMG160 | *Sal*I/*Xba*I PCR fragment of *tyeA* with a missense mutation at codon 5 (Leu_CTT_→Ala_GCA_) in pDM4, Cm^R^ | This study |
| pJMG161 | *Sal*I/*Xba*I PCR fragment of *tyeA* with a missense mutation at codon 33 (Phe_TTT_→Ala_GCA_) in pDM4, Cm^R^ | This study |
| pJMG162 | *Sal*I/*Xba*I PCR fragment of *yopN* with a missense mutation at codon 279 (Trp_TGG_→Cys_TGC_), a nonsense (‘TAG’) mutation inserted after codon 287 and  *tyeA* with a missense mutation at codon 8 (Phe_TTT_→Cys_TGT_) in pDM4, Cm^R^ | This study |
| pJMG163 | *Sal*I/*Xba*I PCR fragment of *tyeA* with a missense mutation at codon 3 (Tyr_TAC_→Ala_GCA_) in pDM4, Cm^R^ | This study |
| pJMG164 | *Sal*I/*Xba*I PCR fragment of *tyeA* with a missense mutation at codon 8 (Phe_TTT_→Ala_GCA_) in pDM4, Cm^R^ | This study |

**Table S3| Oligonucleotides used i**n this study

| **Purpose/vector** | **Oligonucleotide name and sequence** |
| --- | --- |
| *In cis* site-directed mutagenesis | |
| pSF020 (*yopN*_Δ248-272_) | pyopNa2, 5´-AAA *GTC GAC* CTG CGG CTT GCG TGA TG-3´ (*Sal*I) and pN248-272b, 5´-TTG TTG ACT TTG TAG ATC TGC-3´  pN248-272c, 5´-GAT CTA CAA AGT CAA CAA GAC CAA GTT AAA GGA TTT TGG C-3´ and ptyeAd, 5´-AAA *TCT AGA* GAA CGC GCT AAC CAC-3´ (*Xba*I) |
| pAA250 (*yopN*_288(scramble)293_) | pyopNa2 (*Sal*I) and pyopN-22.1b, 5´-GAC AAA TCG TAA GCC ATA AGT TTT ACC CTC TG-3´  pyopN-22.1c, 5´-TAT GGC TTA CGA TTT GTC TGA GTT TAT GGG AGA T-3´ and ptyeAd (*Xba*I) |
| pAA245 (*yopN*_279(F+1), 287(F−1)_) | pyopNa2 (*Sal*I) and pyopN-19.1b, 5´-GAA AAA AAT TGC CAA ATC CTT TAA CTT GGT C-3´  pyopN-19.1c, 5´-GGA TTT GGC AAT TTT TTT CAG AGG GTA AAA CTA AAT GGC GTA CGA CC-3´ and ptyeAd (*Xba*I) |
| pAA244 (*yopN*_279(F+1), 287STOP_) | pyopNa2 (*Sal*I) and pyopN-19.1b  pyopNstop19c, 5´-GGA TTT GGC AAT TTT TTT CAG AGG GTA AAA TGA ATG GCG TAC GAC C-3´ and ptyeAd (*Xba*I) |
| pAA249 (*yopN*_288STOP_) | pyopNa2 (*Sal*I) and pyopN-19.2b, 5´-CTG AAA AAA ATT GCT AAA ATC CTT TAA CTT GGT C-3´  pyopN-19.2c, 5´-GGA TTT TAG CAA TTT TTT CAG AGG GT-3´ and ptyeAd (*Xba*I) |
| pAA246 (*yopN*_279STOP_) | pyopNa2 (*Sal*I) and pyopNstopb2, 5´-CCA TCT AAG TTT TAC CCT CTG AAA AAA ATT GCC-3´  pyopNstopc2, 5´-CAG AGG GTA AAA CTT AGA TGG CGT ACG ACC TTT CTG AG-3´ and ptyeAd (*Xba*I) |
| pJMG159 (*yopN*_W79G_) | pyopNa1, 5´-AAA *GTC GAC* ACC TAC AAT GCC ATG ACG-3’ (*Sal*I) and pyopN-W279Gb, 5´-GAA AAA AAT TGG CCA AAT CCT TTA ACT TGG TC-3´  pyopN-W279Gc, 5´-GGA TTT GGC CAA TTT TTT TCA GAG GGT AAA ACT-3´ and ptyeAd (*Xba*I) |
| pJMG163 (*tyeA*_Y3A_) | ptyeAd20-59a, 5’ –AAG G*CT CGA G*GG ATA TAG ACT CGG TGA-3’ (*Xho*I) and ptyeA-Y3Ab, 5´-GAA AGG TCT GCC GCC ATT AGT TTT ACC CTC TG-3´  ptyeA-Y3Ac, 5´-CTA ATG GCG GCA GAC CTT TCT GAG TTT ATG GGA G-3´ and ptyeAd20-59d, 5’ –AAG G*TC TAG A*GC GCT AAC CAC AAT GTC A-3’ (*Xba*I) |
| pJMG160 (*tyeA*_L5A_) | ptyeAd20-59a (*Xho*I) and ptyeA-L5Ab, 5´-CTC AGA TGC GTC GTA CGC CAT TAG TTT TAC CCT C-3´  ptyeA-L5Ac, 5´-GCG TAC GAC GCA TCT GAG TTT ATG GGA GAT-3´ and ptyeAd20-59d (*Xba*I) |
| pJMG164 (*tyeA*_F8A_) | ptyeAd20-59a (*Xho*I) and ptyeA-F8Ab, 5´-TCC CAT TGC CTC AGA AAG GTC GTA CGC CA-3´  ptyeA-F8Ac, 5´-CTT TCT GAG GCA ATG GGA GAT ATT GTC GCA C-3´ and ptyeAd20-59d (*Xba*I) |
| pJMG161 (*tyeA*_F33A_) | ptyeAd20-59a (*Xho*I) and ptyeA-F33Ab, 5´-AGG AAG GGA TGC GGC GTT GGC AAG ATG TTC-3´  ptyeA-F33Ac, 5´-AAC GCC GCA TCC CTT CCT ACG CCT GAA ATC-3´ and ptyeAd20-59d (*Xba*I) |
| *In trans* site-directed mutagenesis | |
| BACTH | |
| pAA203 & pAA204 (*yopN*) | pNterm-yopN(Xb), 5´-ACG *TCT AGA* GAC GAC GCT TCA TAA CCT ATC-3´ (*Xba*I) and pNterm-yopN(Ec), 5´-ACG *GAA TTC* TCA GAA AGG TCG TAC GCC ATT AG-3´ (*Eco*RI) |
| pAA202 & pAA205 (*yopN*) | pNterm-yopN(Xb) (*Xba*I) and pCterm-yopN(Ec), 5´-ACG *GAA TTC* TTG AAA GGT CGT ACG CCA TTA G-3´ (*Eco*RI) |
| pMF911 (*yopN*_279(F+1), 287STOP_) | pNterm-yopN(Xb) (*Xba*I) and pNterm-19(Ec), 5´-ACG *GAA TTC* CAT TCA TTT TAC CCT CTG AA-3´ (*Eco*RI) |
| pMF912 (*yopN*_279(F+1), 287(F−1)_) | pNterm-yopN(Xb) (*Xba*I) and pNterm-19.1(Ec), 5´-ACG *GAA TTC* TCA GAA AGG TCG TAC GCC A-3´ (*Eco*RI) |
| pMF913 (*yopN*_279STOP_) | pNterm-yopN(Xb) (*Xba*I) and pNterm-19.2(Ec), 5´-ACG *GAA TTC* CTA AAA TCC TTT AAC TTG GTC-3´ (*Eco*RI) |
| pMF914 (*yopN*_288STOP_) | pNterm-yopN(Xb) (*Xba*I) and pNterm-22(Ec), 5´-ACG *GAA TTC* CAT CTA AGT TTT ACC CTC TGA A-3´ (*Eco*RI) |
| pMF915 (*yopN*_288(scramble)293_) | pNterm-yopN(Xb) (*Xba*I) and pNterm-22.1(Ec), 5´-ACG GAA TTC TCA GAC AAA TCG TAA GCC AT-3´ (*Eco*RI) |
| pJMG128 (*yopN*_W279G_) | pNterm-yopN(Xb) (*Xba*I) and pNterm-yopN(Ec) (*Eco*RI) |
| pJMG129 (*yopN*_W279F_) | pNterm-yopN(Xb) (*Xba*I) and pNterm-yopN(Ec) (*Eco*RI) |
| pJMG130 (*yopN*_Δ279_) | pNterm-yopN(Xb) (*Xba*I) and pNterm-yopN(Ec) (*Eco*RI) |
| pMF917 (*yopN*_Δ248-272_) | pNterm-yopN(Xb) (*Xba*I) and pNterm-yopN(Ec) (*Eco*RI) |
| pAA209 & pAA210 (*tyeA*) | pNterm-tyeA(Xb), 5´-ACG *TCT AGA* GGC GTA CGA CCT TTC TGA G-3´ (*Xba*I) and pNterm-tyeA(Ec), 5´-ACG *GAA TTC* TCA ATC CAA CTC ACT CAA TTC-3´ (*Eco*RI) |
| pAA208 & pAA211 (*tyeA*) | pNterm-tyeA(Xb) (*Xba*I) and pCterm-tyeA(Ec), 5´-ACG *GAA TTC* TTA TCC AAC TCA CTC AAT TCT TCC-3´ (*Eco*RI) |
| pJMG131 (*tyeA*_Y3A_) | pNterm-tyeAY3A(Xb), 5´-ACG *TCT AGA* GGC GGC AGA CCT TTC TGA G-3´ (*Xba*I) and pCterm-tyeA(Ec) (*Eco*RI) |
| pJMG134 (*tyeA*_L5A_) | pNterm-tyeAL5A(Xb), 5´-ACG *TCT AGA* GGC GTA CGA CGC ATC TGA G-3´ (*Xba*I) and pCterm-tyeA(Ec) (*Eco*RI) |
| pJMG132 (*tyeA*_F8A_) | pNterm-tyeA(Xb) (*Xba*I) and pCterm-tyeA(Ec) (*Eco*RI) |
| pJMG133 (*tyeA*_F33A_) | pNterm-tyeA(Xb) (*Xba*I) and pCterm-tyeA(Ec) (*Eco*RI) |
| Yeast two hybrid | |
| pAA200 & pAA201 (*yopN*) | pY2HyopN(Ec), 5´-ACG *GAA TTC* ATG ACG ACG CTT CAT AAC CT-3´ (*Eco*RI) and pY2HyopN(Bm), 5´-ACG *GGA TCC* TCA GAA AGG TCG TAC GCC ATT AG-3´ (*Bam*HI) |
| pJMG080 (*yopN*_279(F+1), 287STOP_) | pY2HyopN(Ec) (*Eco*RI) and pY2HyopN-19(Bm), 5´-ACG *GGA TCC* ATT CAT TTT ACC CTC TGA A-3´ (*Bam*HI) |
| pJMG081 (*yopN*_279(F+1), 287(F−1)_) | pY2HyopN(Ec) (*Eco*RI) and pY2HyopN-19.1(Bm), 5´-ACG *GGA TCC* TCA GAA AGG TCG TAC GCC A-3´ (*Bam*HI) |
| pJMG082 (*yopN*_279STOP_) | pY2HyopN(Ec) (*Eco*RI) and pY2HyopN-19.2(Bm), 5´-ACG *GGA TCC* CTA AAA TCC TTT AAC TTG GTC-3´ (*Bam*HI) |
| pJMG083 (*yopN*_288STOP_) | pY2HyopN(Ec) (*Eco*RI) and pY2HyopN-22(Bm), 5´-ACG *GGA TCC* ATC TAA GTT TTA CCC TCT GA-3´ (*Bam*HI) |
| pJMG084 (*yopN*_288(scramble)293_) | pY2HyopN(Ec) (*Eco*RI) and pY2HyopN-22.1(Bm), 5´-ACG *GGA TCC* TCA GAC AAA TCG TAA GCC AT-3´ (*Bam*HI) |
| pJMG121 (*yopN*_W279G_) | pY2HyopN(Ec) (*Eco*RI) and pY2HyopN(Bm) (*Bam*HI) |
| pJMG122 (*yopN*_W279F_) | pY2HyopN(Ec) (*Eco*RI) and pY2HyopN(Bm) (*Bam*HI) |
| pJMG123 (*yopN*_Δ279_) | pY2HyopN(Ec) (*Eco*RI) and pY2HyopN(Bm) (*Bam*HI) |
| pJMG086 (*yopN*_Δ248-272_) | pY2HyopN(Ec) (*Eco*RI) and pY2HyopN(Bm) (*Bam*HI) |
| pAA206 & pAA207 (*tyeA*) | pY2HtyeA(Ec), 5´-ACG *GAA TTC* ATG GCG TAC GAC CTT TCT GAG-3´ (*Eco*RI) and pY2HtyeA(Bm), 5´-ACG *GGA TCC* TCA ATC CAA CTC ACT CAA TTC-3´ (*Bam*HI) |
| pJMG124 (*tyeA*_Y3A_) | pY2HtyeA-Y3A(Ec), 5´-ACG *GAA TTC* ATG GCG GCA GAC CTT TCT GAG-3´ (*Eco*RI) and pY2HtyeA(Bm) (*Bam*HI) |
| pJMG125 (*tyeA*_L5A_) | pY2HtyeA-L5A(Ec), 5´-ACG *GAA TTC* ATG GCG TAC GAC GCA TCT GAG-3´ (*Eco*RI) and pY2HtyeA(Bm) (*Bam*HI) |
| pJMG126 (*tyeA*_F8A_) | pY2HtyeA(Ec) (*Eco*RI) and pY2HtyeA(Bm) (*Bam*HI) |
| pJMG127 (*tyeA*_F33A_) | pY2HtyeA(Ec) (*Eco*RI) and pY2HtyeA(Bm) (*Bam*HI) |

**
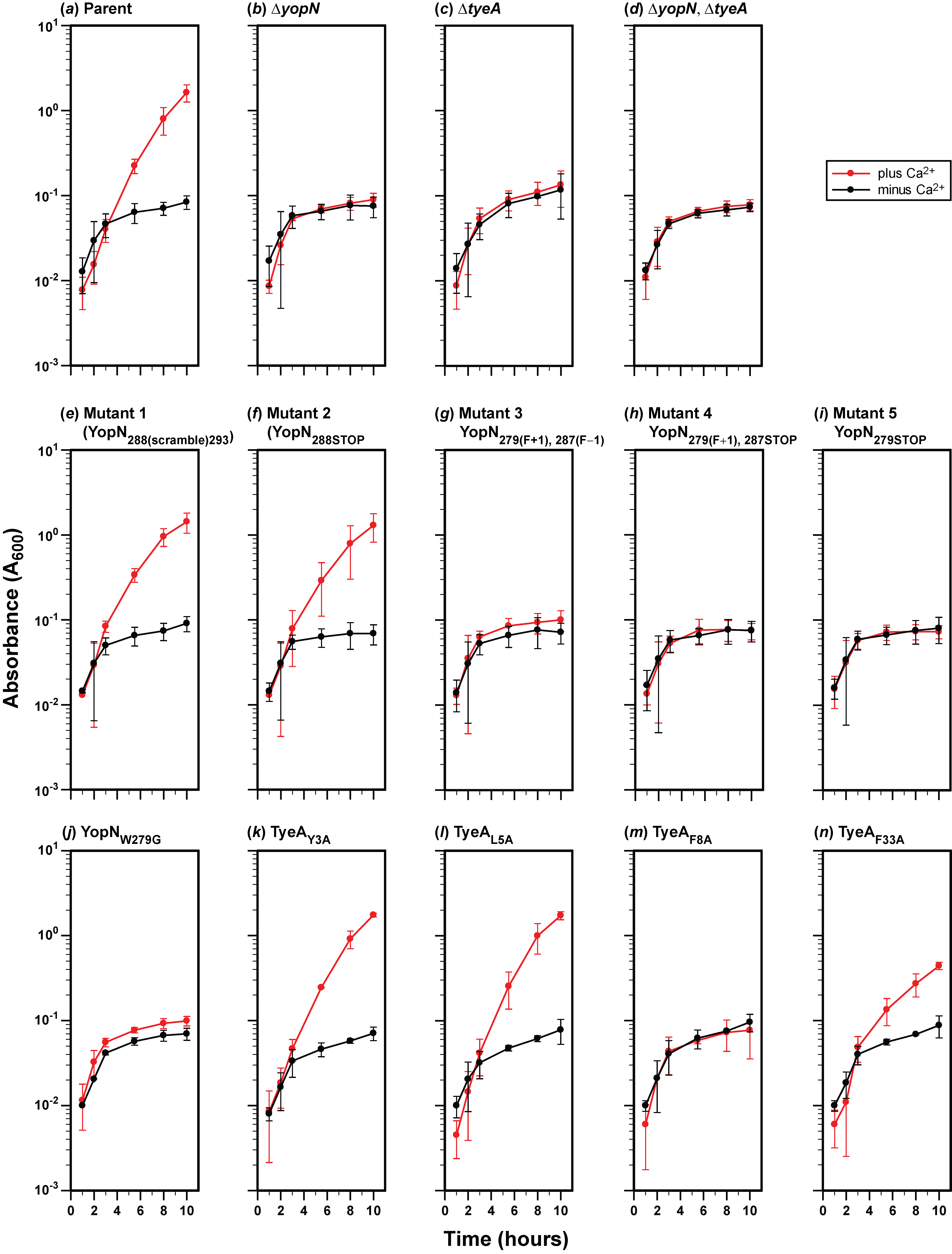
**

**Figure S1| Low calcium dependent growth of *yopN* and *tyeA* mutants.** Bacteria were grown at 37°C in TMH medium alone (minus Ca^2+^; black circles) or supplemented with 2.5 mM CaCl_2_ (plus Ca^2+^; red circles). Two well-characterised growth phenotypes were detected: CD – calcium dependent growth (*a, e, f, k* and *l*), and TS – bacteria are sensitive to elevated temperature regardless of the presence or absence of calcium (*b, c, d, g, h, i, j,* and *m*). A notable outlier was the CD-like intermediate growth phenotype (*n*). Strains: *a*) Parent (YopN_native_, TyeA_native_), YPIII/pIB102; *b*) Δ*yopN* null mutant, YPIII/pIB82; *c*) Δ*tyeA* null mutant, YPIII/pIB801a; *d*) Δ*yopN, tyeA* double mutant, YPIII/pIB8201a; *e*) Mutant 1 - YopN_288(scramble)293_, YPIII/pIB8213; *f*) Mutant 2 - YopN_288STOP_, YPIII/pIB8212; *g*) Mutant 3 - YopN_279(F+1), 287(F−1)_, YPIII/pIB8208; *h*) Mutant 4 - YopN_279(F+1), 287STOP_, YPIII/pIB8207; *i*) Mutant 5 - YopN_279STOP_, YPIII/pIB8209; *j*) YopN_W279G_, YPIII/pIB8223; *k*) TyeA_Y3A_, YPIII/pIB8221; *l*) TyeA_L5A_, YPIII/pIB8222; *m*) TyeA_F8A_, YPIII/pIB8220; *n*) TyeA_F33A_, YPIII/pIB8219.

**
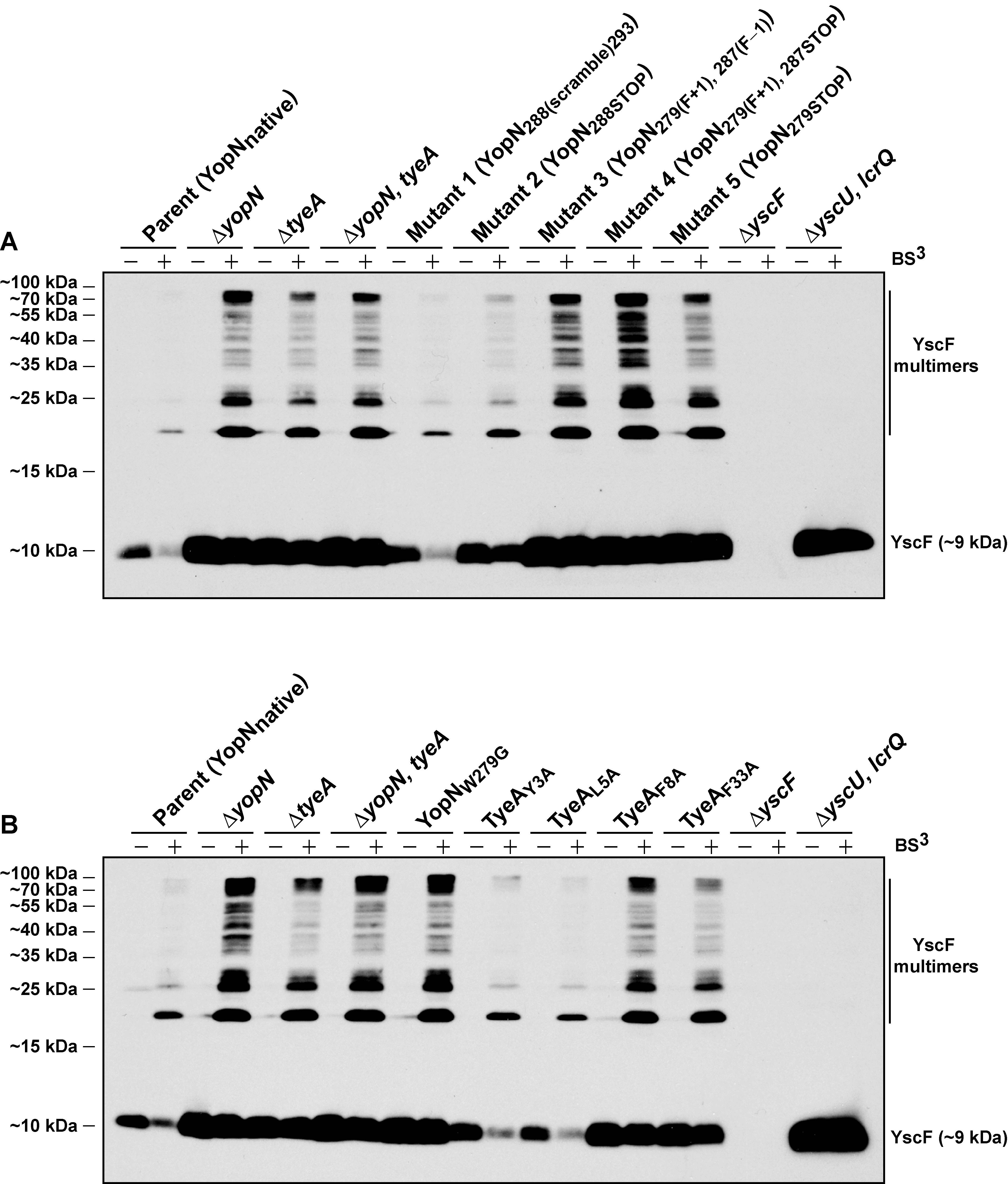
**

**Figure S2| Chemically cross-linked surface-located YscF.** Yersinia strains were grown in non-permissive T3S media (plus Ca^2+^). Where indicated (+), the membrane-impermeable chemical cross-linker BS^3^ was added to the bacteria. After being quenched with Tris-HCl, bacteria pellets were solubilized in sample buffer and then protein fractionated by 12% acrylamide SDS-PAGE. After wet-transfer to PVDF, YscF was detected with immune-absorbed monospecific anti-YscF antiserum. Non-cross-linked monomeric YscF was observed in all lanes except the Δ*yscF* null mutant control. Cell-surface YscF multimers were observed in all lanes except for the Δ*yscF* null mutant control as well as the YscF^+^, but T3SS-defective, Δ*yscU, lcrQ* null mutant control. The predicted molecular mass of monomeric YscF is given in parenthesis, while approximate sizes of protein molecular weight standards are given to the right. Strains (A): Parent (YopN_native_), YPIII/pIB102; Δ*yopN* null mutant*,* YPIII/pIB82; Δ*tyeA* null mutant*,* YPIII/pIB801a; Δ*yopN, tyeA* double mutant, YPIII/pIB8201a; Mutant 1 - YopN_288(scramble)293_, YPIII/pIB8213; Mutant 2 - YopN_288STOP_, YPIII/pIB8212; Mutant 3 - YopN_279(F+1), 287(F−1)_, YPIII/pIB8208; Mutant 4 - YopN_279(F+1), 287STOP_, YPIII/pIB8207; Mutant 5 - YopN_279STOP_, YPIII/pIB8209; Δ*yscF* null mutant, YPIII/pIB202; Δ*yscU, lcrQ* double mutant, YPIII/pIB75-26. Strains (B): Parent (YopN_native_), YPIII/pIB102; Δ*yopN* null mutant*,* YPIII/pIB82; Δ*tyeA* null mutant*,* YPIII/pIB801a; Δ*yopN, tyeA* double mutant, YPIII/pIB8201a; YopN_W279G_, YPIII/pIB8223; TyeA_Y3A_, YPIII/pIB8221; TyeA_L5A_, YPIII/pIB8222; TyeA_F8A_, YPIII/pIB8220; TyeA_F33A_, YPIII/pIB8219; Δ*yscF* null mutant, YPIII/pIB202; Δ*yscU, lcrQ* double mutant, YPIII/pIB75-26.

**
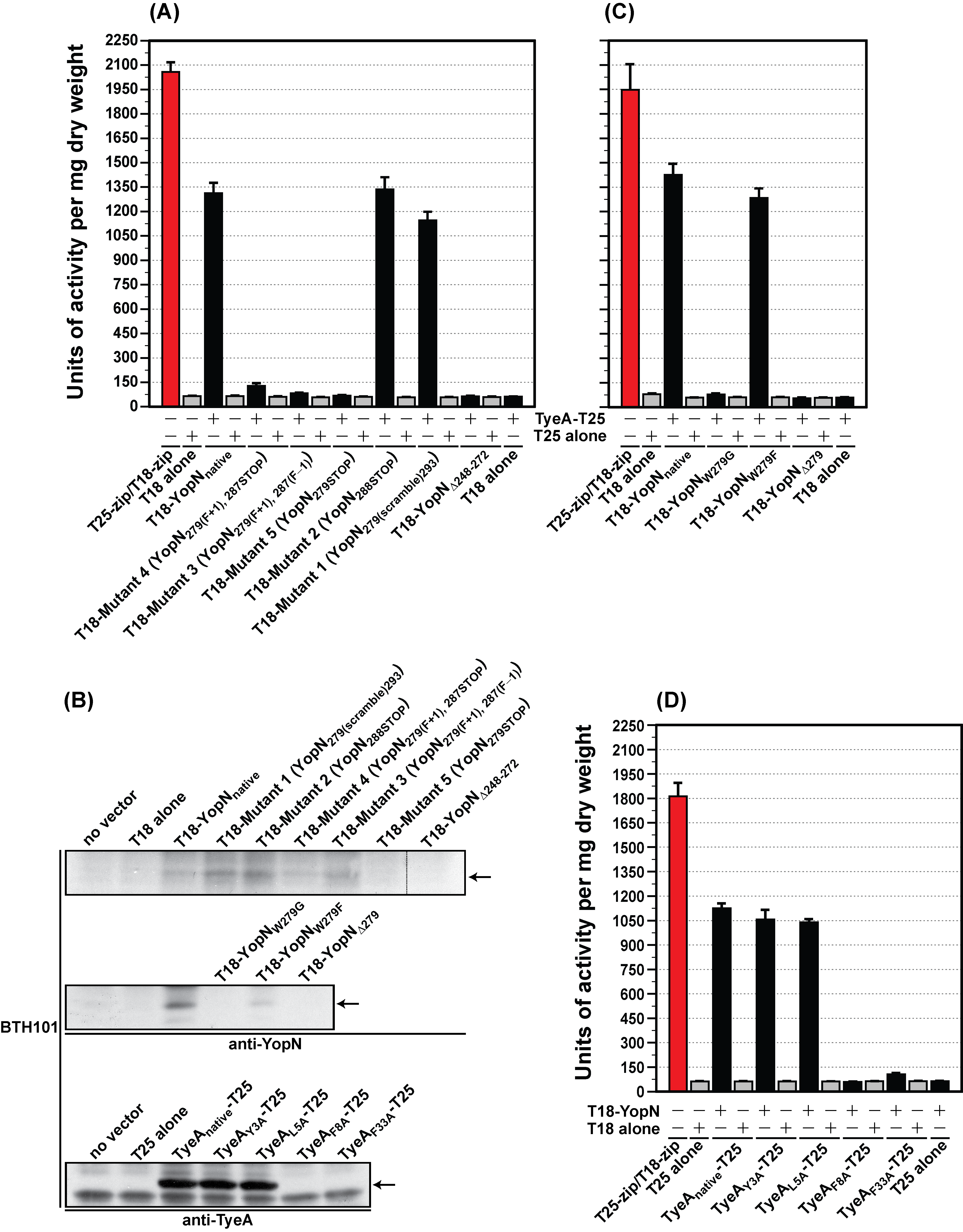
**

**Figure S3| Interaction analysis of YopN and TyeA fusions used in the BACTH assay.** Native and mutated *yopN* alleles were translationally fused to the C-terminus of CyaA_225-399_ (T18) in the vector pUT18C (to give T18-YopN_native_ and the various mutated equivalents). Native and mutated *tyeA* alleles were translationally fused to the N-terminus of CyaA_1-224_ (T25) in the vector pKNT25 (to give TyeA_native_-T25 and mutated equivalents). BACTH interaction analysis of pairwise combinations of T25 and T18 hybrids was quantified via measurement of β-galactosidase activity and is represented as units/mg dry weight of host *E. coli* BTH101 bacteria (*a, c* and *d*). As an internal positive control, we used the constructs provided by the manufacture that expressed T18-Zip (vector pUT18C-Zip) and T25-Zip (vector pKT25-Zip). Internal negative controls used the appropriate combinations of empty BATCH expression vectors *i.e.*: expressing only T18 (pUT18 or pUT18C) and/or T25 (pKT25 or pKNT25) alone. As in previous studies , a level of β-galactosidase activity at least 3-fold higher than the appropriate negative control was considered to indicate a significant positive interaction. Data is presented as the mean (± standard error of the mean) of at least four independent experiments performed in triplicate. To examine for stable expression of each variant in BTH101, protein extracts were generated from bacteria and separated by SDS-PAGE as described previously . The YopN and TyeA fusions were identified by immunoblot analysis using anti-YopN and anti-TyeA antibody, respectively (*b*). In each case, a protein extract from BTH101 harboring the relevant vector alone was included as a negative control. The approximate molecular weight of the T18::YopN variants were predicted to be around 52.0 kDa and the TyeA::T25 variants around 35.0 kDa. Plasmids: T18-YopN_native_, pAA203; T18-Mutant 4 - YopN_279(F+1), 287STOP_, pMF911; T18-Mutant 3 - YopN_279(F+1), 287(F−1)_, pMF912; T18-Mutant 5 - YopN_279STOP_, pMF913; T18-Mutant 2 - YopN_288STOP_, pMF914; T18-Mutant 1 - YopN_288(scramble)293_, pMF915; T18-YopN_Δ248-272_, pMF917; T18-YopN_W279G_, pJMG128; T18-YopN_W279F_, pJMG129; T18-YopN_Δ279W_, pJMG130; TyeA_native_-T25, pAA211; TyeA_Y3A_-T25, pJMG131; TyeA_L5A_-T25, pJMG134; TyeA_F8A_-T25, pJMG132; TyeA_F33A_-T25, pJMG133.

**REFERENCES**

1 Amer, A. A., Costa, T. R., Farag, S. I., Avican, U., Forsberg, A., Francis, M. S. 2013 Genetically Engineered Frameshifted YopN-TyeA Chimeras Influence Type III Secretion System Function in Yersinia pseudotuberculosis. *PLoS One*. **8**, e77767.

2 Amer, A. A., Ahlund, M. K., Broms, J. E., Forsberg, A., Francis, M. S. 2011 Impact of the N-terminal secretor domain on YopD translocator function in Yersinia pseudotuberculosis type III secretion. *J Bacteriol*. **193**, 6683-6700.

3 Thanikkal, E. J., Mangu, J. C., Francis, M. S. 2012 Interactions of the CpxA sensor kinase and cognate CpxR response regulator from Yersinia pseudotuberculosis. *BMC Res Notes*. **5**, 536.

4 Simon, R., Priefer, U., Pühler, A. 1983 A broad host range mobilisation system for in vivo genetic engineering: transposon mutagenesis in Gram negative bacteria. *Nature Biotechnology*. **1**, 784-791.

5 Lavander, M., Sundberg, L., Edqvist, P. J., Lloyd, S. A., Wolf-Watz, H., Forsberg, Å. 2002 Proteolytic cleavage of the FlhB homologue YscU of Yersinia pseudotuberculosis is essential for bacterial survival but not for type III secretion. *J Bacteriol*. **184**, 4500-4509.

6 Rosqvist, R., Forsberg, Å., Rimpiläinen, M., Bergman, T., Wolf-Watz, H. 1990 The cytotoxic protein YopE of Yersinia obstructs the primary host defence. *Mol Microbiol*. **4**, 657-667.

7 Karimova, G., Dautin, N., Ladant, D. 2005 Interaction network among Escherichia coli membrane proteins involved in cell division as revealed by bacterial two-hybrid analysis. *J Bacteriol*. **187**, 2233-2243.
